# Gut bacteria-derived 5-hydroxyindole is a potent stimulant of intestinal motility via its action on L-type calcium channels

**DOI:** 10.1371/journal.pbio.3001070

**Published:** 2021-01-22

**Authors:** Barbora Waclawiková, Amber Bullock, Markus Schwalbe, Carmen Aranzamendi, Sieger A. Nelemans, Gertjan van Dijk, Sahar El Aidy

**Affiliations:** 1 Host-Microbe Metabolic Interactions, Groningen Biomolecular Sciences and Biotechnology Institute (GBB), University of Groningen, Groningen, the Netherlands; 2 Department of Molecular Neurobiology, Groningen Institute for Evolutionary Life Sciences (GELIFES), University of Groningen, Groningen, the Netherlands; 3 Department of Behavioral Neurosciences, Cluster Neurobiology, Groningen Institute of for Evolutionary Life Sciences (GELIFES), University of Groningen, Groningen, The Netherlands; New York University School of Medicine, UNITED STATES

## Abstract

Microbial conversion of dietary or drug substrates into small bioactive molecules represents a regulatory mechanism by which the gut microbiota alters intestinal physiology. Here, we show that a wide variety of gut bacteria can metabolize the dietary supplement and antidepressant 5-hydroxytryptophan (5-HTP) to 5-hydroxyindole (5-HI) via the tryptophanase (TnaA) enzyme. Oral administration of 5-HTP results in detection of 5-HI in fecal samples of healthy volunteers with interindividual variation. The production of 5-HI is inhibited upon pH reduction in in vitro studies. When administered orally in rats, 5-HI significantly accelerates the total gut transit time (TGTT). Deciphering the underlying mechanisms of action reveals that 5-HI accelerates gut contractility via activation of L-type calcium channels located on the colonic smooth muscle cells. Moreover, 5-HI stimulation of a cell line model of intestinal enterochromaffin cells results in significant increase in serotonin production. Together, our findings support a role for bacterial metabolism in altering gut motility and lay the foundation for microbiota-targeted interventions.

## Introduction

The gastrointestinal (GI) tract is home to trillions of microbes. The gut microbiota produces a wide range of small bioactive molecules derived from various substrates, including dietary precursors and medications [1,2]. Such microbial conversion represents a significant regulatory mechanism by which gut microbes can alter intestinal host physiology, including gut motility [3,4]. For example, tryptamine produced by bacterial decarboxylation of dietary tryptophan accelerates GI transit by activating epithelial G-protein coupled receptor, serotonin receptor 4, and increasing anion-dependent fluid secretion in the proximal colon of mice [5]. Therefore, gut microbiota-derived molecules appear to functionally link the microbiota activity to the host gut motility.

Gut motility is a tightly controlled system involving the main excitatory neurotransmitter, acetylcholine (ACh), whose excitatory effect on the intestinal smooth muscle is mediated through the muscarinic ACh receptors [6]. Stimulation of the muscarinic receptors by ACh induces contractions that depend on voltage-dependent and voltage-independent Ca^2+^ entry and intracellular Ca^2+^ release [7] (cholinergic neurotransmission and smooth muscle biology is extensively discussed in [6,8]). Serotonin (5-HT) is a neurochemical, which has been implicated in the control of gut motility [9,10]; however, the functional role of endogenous 5-HT remains mysterious, since depletion of neuronal or mucosal 5-HT has little or no effect on gut motility [11,12]. On the other hand, the role of endogenous 5-HT produced from enterochromaffin cells in regulating gut motility remains obscure [9]. However, it has been described that endogenous 5-HT can act as a modulator of intestinal motility via activation of 5-HT_3_ and 5-HT_4_ receptors in the enteric nervous system (ENS) (13). Within this tightly controlled system, disturbances in these regulatory mechanisms have been associated with gut motility disorders.

Constipation is a common, debilitating motility disorder affecting up to 27% of the population [14]. Constipation is also often associated with colorectal cancer, Parkinson disease, childhood attention-deficit/hyperactivity disorder, and autism spectrum disorder, as well as mood disorders [15–19]. Recently, the administration of 5-hydroxytryptophan (5-HTP) to a mouse model of depression resulted in a normalized total GI transit time and increased colonic motility [20]. Therefore, 5-HTP can be a potential novel treatment for patients with intestinal motility dysfunction associated with mood disorders [20].

5-HTP (also known as oxitriptan) is a naturally occurring amino acid, as well as a chemical precursor and intermediate metabolite of the essential amino acid L-tryptophan in the biosynthesis of serotonin [21]. 5-HTP is used as a food supplement or as a drug with or without other medications for the treatment of a wide variety of conditions, including depression, fibromyalgia, binge eating associated with obesity, chronic headaches, and insomnia [22–27]. 5-HTP is a structural homologue of L-tryptophan, which is metabolized by gut bacterial tryptophanase (TnaA) enzyme to produce indole [28]. However, it remains unknown whether gut bacteria can also metabolize 5-HTP and whether the produced products can modulate GI motility.

Here, we describe how 5-HTP is metabolized by gut bacteria. We show that a wide variety of gut bacteria convert 5-HTP to 5-hydroxyindole (5-HI) and that the degree of this conversion is highly dependent on the microbial composition and changes in the pH. Moreover, our findings reveal 5-HI to be a potent stimulator of gut contractility both ex vivo and in vivo.

## Results

### Gut bacteria convert 5-hydroxytryptophan to 5-hydroxyindole

**Fig 1:**
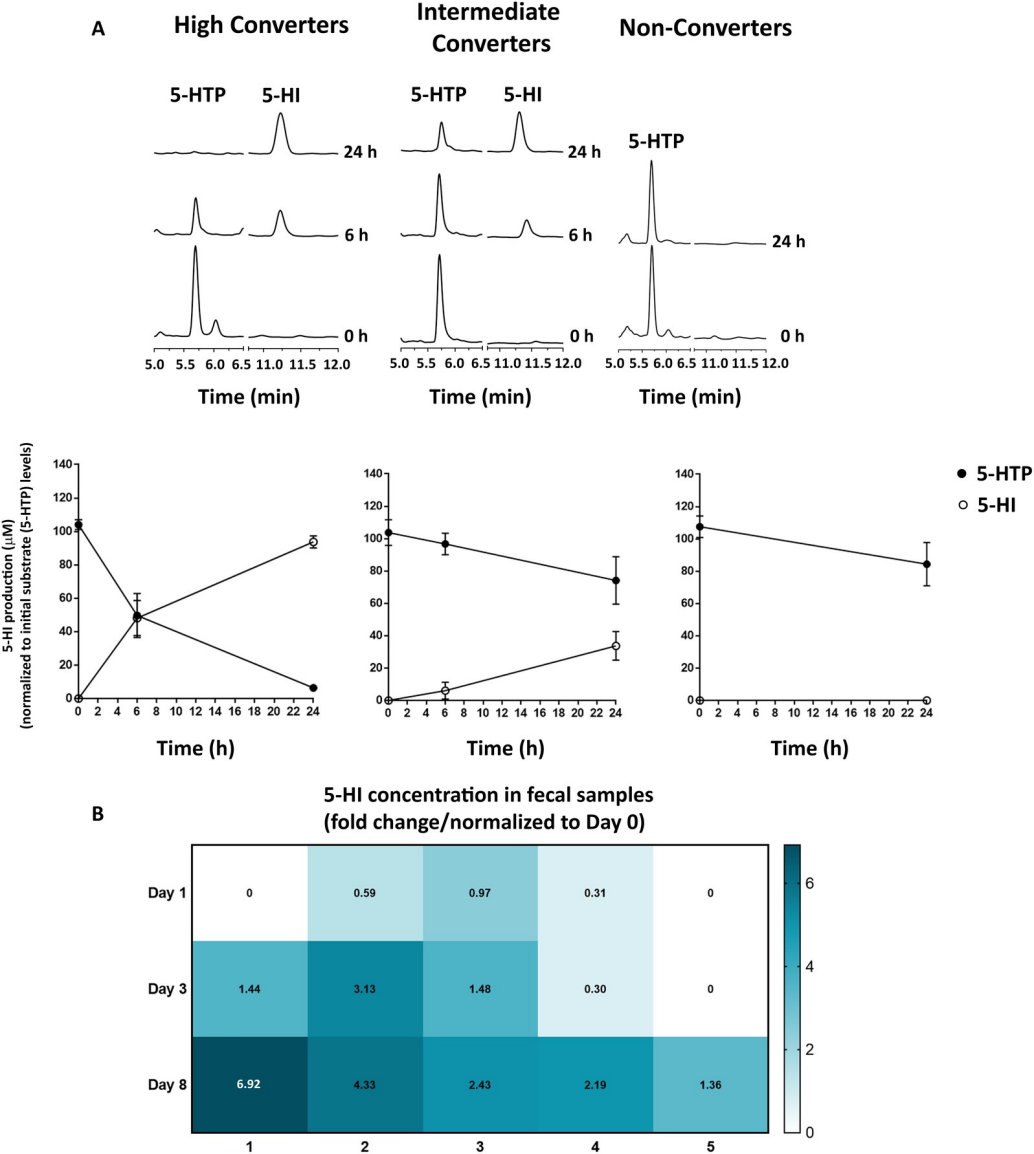
Gut bacteria convert 5-HTP to 5-HI. **(A)** From left to right: chromatograms showing bacterial conversion of 100 μM 5-HTP to 5-HI after 6 and 24 h of incubation of human fecal samples (upper panel). Lower panel: quantification of 5-HTP consumption (substrate; black circles) and 5-HI production (product; white circles) observed in High (*n* = 8), Intermediate (*n* = 4), and Non-Converters (*n* = 6). The raw data can be found in S1 Raw images. (**B)** Heatmap of 5-HI levels detected in the fecal samples of volunteers who administered 5-HTP tablets for 1 week, collected on Day 1 (first ingestion of 5-HTP tablet), Day 3, and Day 8 (1 day after last 5-HTP tablet was ingested). The numbers in the squares indicate fold change in 5-HI levels normalized to Day 0. The raw data used for quantification of **A** (lower panel) and **B** can be found in S1 Data. 5-HI, 5-hydroxyindole; 5-HTP, 5-hydroxytryptophan.

5-HTP is absorbed throughout the entire GI tract [29,30] when taken as a food supplement or as an antidepressant. Upon absorption, 5-HTP is partially converted into 5-HT before it reaches the brain [20]. To determine whether gut bacteria have the ability to metabolize 5-HTP before it is taken up by the intestinal tissue, human fecal samples from healthy volunteers (*n* = 18) were incubated anaerobically ex vivo with 100 μM 5-HTP and analyzed by High-Performance Liquid Chromatography coupled with Electrochemical detection and UV detection (HPLC-ED/UV). Chromatograms revealed the formation of an unknown peak (**Fig 1A**), which was further identified by Liquid Chromatography-Mass Spectrometry (LC-MS) to be 5-HI (**S1A Fig**). Interestingly, there was a variation among the tested fecal samples in their ability to convert 5-HTP into 5-HI, ranging from samples that either completely or partially metabolized 5-HTP to 5-HI (High and Intermediate Converters; *n* = 8 and 4, respectively), to samples where 5-HTP was not metabolized at all (Non-Converters; *n* = 6) (**Fig 1A,** upper panel). Quantification of 5-HI production within the High and Intermediate Converters showed approximately 50% and 10% of 5-HTP metabolized after 6 h, respectively. After 24 h, 5-HTP was fully metabolized in the High Converters and partially metabolized (approximately 30%) in the Intermediate Converters (**Fig 1A,** lower panel). No other metabolites were detected in the 5-HTP treated samples, suggesting that the gut microbiota metabolize 5-HTP only for the production of 5-HI. Notably, no basal levels of 5-HTP were detected by HPLC-ED/UV in the control samples (**S1B Fig;** 0 h time point). Similarly, no indole was detected in the Non-Converters samples, excluding the fact that tryptophan might be blocking the conversion of 5-HTP (**S1C Fig**).

To further support the possibility that 5-HTP is metabolized in the gut lumen before it is absorbed in the gut tissue where it is converted into 5-HT, 5 healthy volunteers were randomly selected out of the 18 participants, who participated in the fecal samples’ donation. Participants were orally administered 5-HTP tablets (Swanson Health Products, Fargo, North Dakota, United States of America) (50 mg 5-HTP per tablet) daily for 1 week, as recommended by the manufacturer. Fecal samples were collected for targeted metabolomic analysis on Day 0 (control, no 5-HTP ingested), Day 1 (first ingestion of 5-HTP tablet), Day 3, and Day 8 (1 day after last 5-HTP tablet was ingested), respectively. Targeted metabolomic analysis revealed that the levels of 5-HI in the fecal samples gradually increased during the period of 5-HTP intake **(Fig 1B)**. Interestingly, there was a similar variation among the tested participants in their ability to convert 5-HTP into 5-HI as observed in the ex vivo screening (**Fig 1A**). Taken together, the results show that gut bacteria can metabolize 5-HTP with interindividual variation in their ability to produce 5-HI.

### Bacterial tryptophanase is responsible for the conversion of 5-hydroxytryptophan

**Fig 2:**
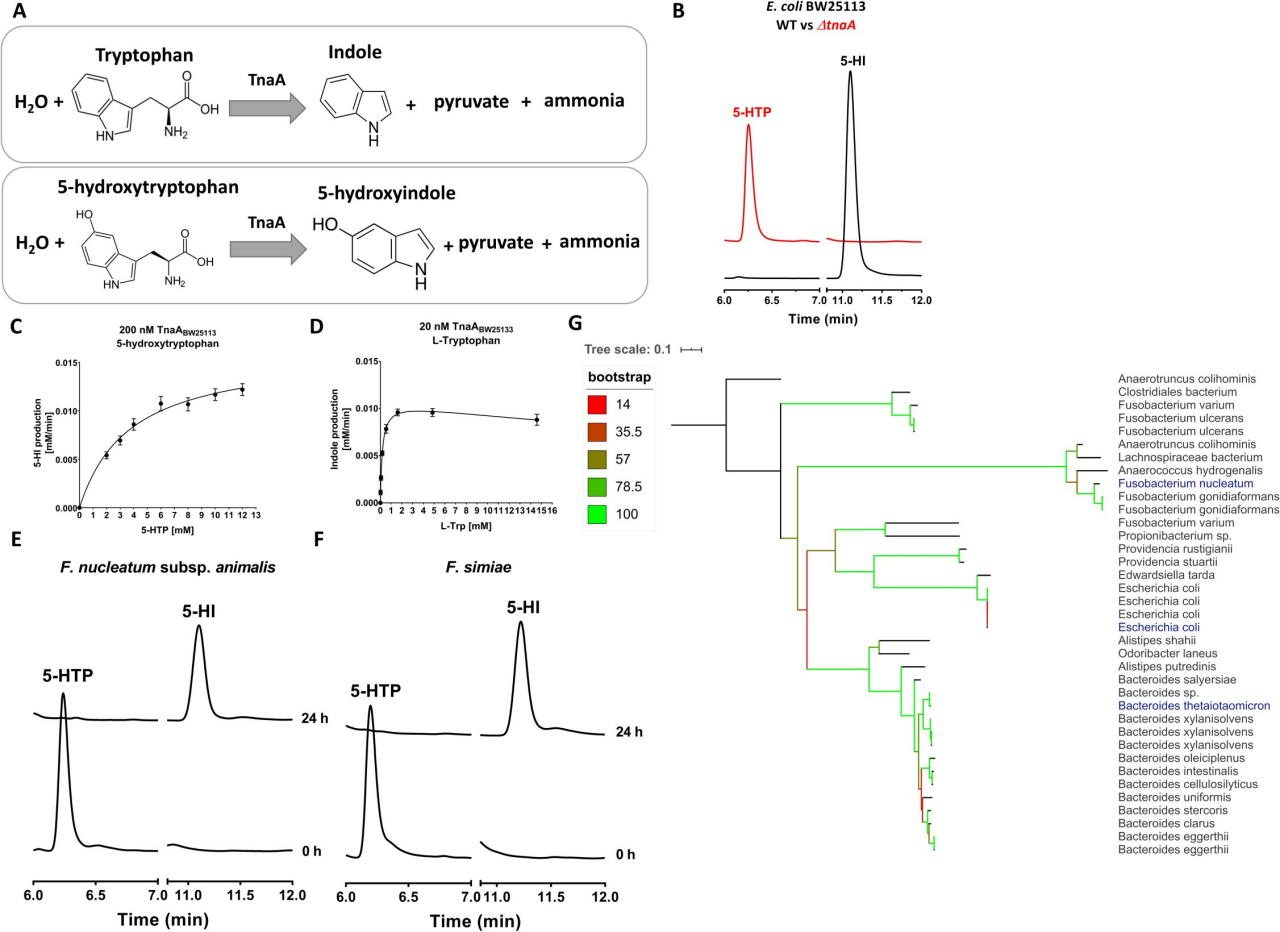
Bacterial tryptophanase is responsible for the conversion of 5-HTP. **(A)** β-elimination reaction for tryptophan and 5-HTP. (**B)** Overnight culture of *E*. *coli* BW25113^WT^ (black line) and *E*. *coli* BW25113^*ΔtnaA*^ (red line) incubated at 37 °C with agitation with 100 μM 5-HTP for 24 h. Curves represent 1 example of 3 biological replicates. Only *E*. *coli* BW25113^*ΔtnaA*^ mutant was tested since previous reports showed the same function of TnaA enzyme among different bacterial species [34,35]. The raw data can be found in S2 Raw images. (**C, D)** Michaelis–Menten kinetic curves for (**C)** 5-HTP and (**D)** tryptophan as substrates for 200 nM or 20 nM TnaA_BW25113_, respectively. Reactions were performed in biological triplicate using 5-HTP concentrations ranging from 0 to 12 mM and tryptophan concentrations ranging from 0 to 14.58 mM. Enzyme kinetics were calculated using a nonlinear Michaelis–Menten regression model for 5-HTP and a nonlinear substrate inhibition kinetic model for tryptophan. Error bars represent the SEM. The raw data used for enzyme kinetics of **C** and **D** can be found in S2 Data. (**E, F)** Overnight cultures of *F*. *nucleatum* subsp. *animalis* and *F*. *simiae* incubated anaerobically at 37 °C with 50 μM 5-HTP. Curves are a representative example of 3 biological replicates. The raw data can be found in S2 Raw images. (**G)** Phylogenetic tree created using iTOL online tool showing 15 closest orthologues (indicated in black) to TnaA enzymes from *F*. *nucleatum*, *E*. *coli*, and *B*. *thetaiotamicron* (indicated in blue). 5-HTP, 5-hydroxytryptophan; TnaA, tryptophanase.

Since 5-HI is a structural analogue of indole, which is produced by bacterial degradation of L-tryptophan via TnaA enzyme, we hypothesized that 5-HTP is also a substrate for the TnaA enzyme (28) (**Fig 2A)**. To verify our hypothesis, *E*. *coli* BW25113^*ΔtnaA*^ mutant was incubated with 5-HTP and was compared to the wild-type *E*. *coli* BW25113^*WT*^ strain. Overnight incubation of *E*. *coli* BW25113^*ΔtnaA*^ and *E*. *coli* BW25113^*WT*^ bacterial cells with 5-HTP showed that the production of 5-HI was completely abolished in the mutant strain as analyzed by HPLC-ED/UV (**Fig 2B**), indicating that 5-HTP is degraded to 5-HI by TnaA enzyme. Similarly, the production of indole from tryptophan was eliminated in the mutant strain, further confirming the involvement of TnaA enzyme in both conversions (**S2A Fig**).

To further characterize the substrate specificity and kinetic parameters of the TnaA enzyme, *tnaA* gene from *E*. *coli* BW25113 was expressed in *E*. *coli* BL21 (DE3) and then purified. As expected, Michaelis–Menten kinetics indicated TnaA had a lower affinity (*K*_M_) and catalytic efficiency (*k*_cat_/*K*_M_) for 5-HTP than its natural substrate tryptophan as illustrated by the *K*_M_ and *k*_cat_/*K*_M_ values for 5-HTP (3.7 ± 0.2 mM and 21.9 min^−1^ mM^−1^, respectively) compared to those for tryptophan (0.19 ± 0.009 mM and 2823.7 min^−1^ mM^−1^, respectively) (**Fig 2C and 2D** and **Table 1**). However, despite the differential substrate affinity, incubation of fecal samples with 5-fold higher levels of tryptophan relative to 5-HTP did not prevent the conversion of 5-HTP to 5-HI (**S2B Fig**).

To determine whether other gut-associated bacterial strains containing TnaA enzyme can convert 5-HTP to 5-HI, a screening of representative bacterial strains was performed. Among these bacteria, members of the commensal gut bacteria, *Fusobacterium nucleatum* subsp. *animalis* and *Fusobacterium simiae* displayed a similar ability to fully metabolize 5-HTP into 5-HI after 24 h of anaerobic incubation (**Fig 2E and 2F**). The gut isolate *E*. *coli* DSM 11250 demonstrated production of 5-HI (approximately 30 μM) after 20 min of incubation at 37 °C, which was 6-fold higher than the laboratory strain *E*. *coli* BW25113 (approximately 5 μM). Moreover, production of 5-HI was detected in *Bacteroides thetaiotamicron* cell lysates (**S2C Fig**), suggesting that TnaA from high abundant Bacteroides genus is also able to convert 5-HTP to 5-HI.

To search for possible explanation of the variation in the production of 5-HI among the tested bacterial strains (**Figs 2E and 2F and S2C**), the protein sequences from *E*. *coli* BW25113, *B*. *thetaiotamicron* VPI-5482, and *F*. *nucleatum* subsp. *animalis* ATCC 51191 (accession numbers: AIN34033.1, AAO76599.1, EGQ80312.1) were used as a query to search the translated HMP Reference Genome sequence database (HMRGD). The proteins were considered homologous when the minimal identity percentage was above 30% and query cover was above 90% [31]. The 15 closest (with highest identity, the whole list can be found in **Table A in S1 Table**) orthologues to each of the initial seed sequences were extracted, and a multiple sequence alignment was performed (**S3 Fig**). This analysis identified differences among the level of conservation in the multiple active sites [32] of TnaA enzyme, which could underlie the observed variation between production of 5-HI among different gut bacterial strains tested (**Figs 2E and 2F and S2C**). The alignment further served as an input for phylogenetic tree showing the TnaA encoding orthologues of *E*. *coli* BW25113, *B*. *thetaiotamicron* VPI-5482, and *F*. *nucleatum* subsp. *animalis* ATCC 51191 (**Fig 2G**). Altogether, these results suggest that TnaA is the enzyme involved in 5-HTP conversion to 5-HI, with some degree of variation in its active sites among various microbiota strains, and that this function is encoded by genomes of a variety of gut bacterial species that are abundantly present in both the small and large intestine [33].

**Table 1:** Enzyme kinetics determined by nonlinear Michaelis–Menten regression model for 5-hydroxytryptophan and nonlinear substrate inhibition kinetic model for tryptophan.

Michaelis–Menten kinetic parameters	
5-hydroxytryptophan (pH 7.4, Tryptophanase_*E*.*coli* BW25113_)	L-Tryptophan (pH 7.4, Tryptophanase_*E*.*coli* BW25113_)
E [nM] = 200	E [nM] = 20
*K*_M_ [mM] = 3.7 ± 0.2	*K*_M_ [mM] = 0.19 ± 0.009
*V*_max_ [mM/min] = 0.016 ± 0.0003	*V*_max_ [mM/min] = 0.011 ± 0.0001
*K*_i_ [mM] = n.a.	*K*_i_ [mM] = 69.79 ± 7.4
*k*_cat_ [min^−1^] = 80.9 ± 1.6	*k*_cat_ [min^−1^] = 536.5 ± 6.3
*k*_cat_/*K*_M_ [min^−1^ mM^−1^] = 21.9	*k*_cat_/*K*_M_ [min^−1^ mM^−1^] = 2823.7
R^2^ = 0.83	R^2^ = 0.93

± indicates the standard error of the mean.

### Bacterial production of 5-hydroxyindole is dependent on the microbiota composition and pH levels

**Fig 3:**
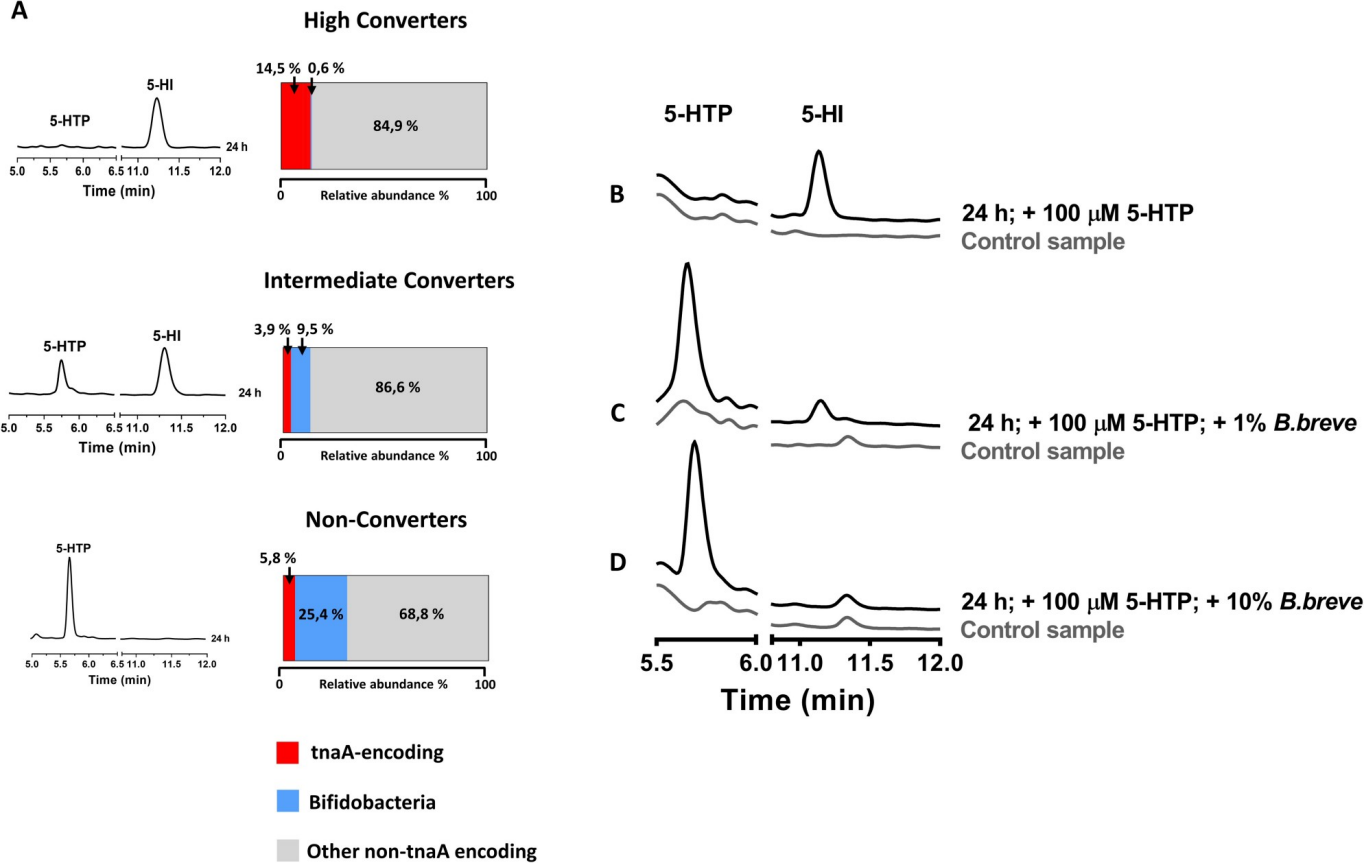
Bacterial production of 5-HI is dependent on the microbiota composition and pH levels. **(A)** Microbial profiling of human fecal samples from High, Intermediate, and Non-Converters shows lower relative abundance of tnaA-encoding gut bacterial genera and higher relative abundance of Bifidobacteria genera, respectively. Chromatograms on the left show variation in conversion of 5-HTP to 5-HI after 24 h of anaerobic incubation with 5-HTP. The raw data can be found in S1 Raw images. (**B–D)** Overnight cultures of *F*. *nucleatum* subsp. *animalis* incubated anaerobically at 37 °C with 100 μM 5-HTP (**B**) in the absence or in the presence of either (**C**) 1% *Bifidobacterium breve* or (**D**) 10% *B*. *breve*. The raw data can be found in S3 Raw images. 5-HI, 5-hydroxyindole; 5-HTP, 5-hydroxytryptophan; tnaA, tryptophanase.

To determine the possible cause of variation in the bacterial conversion of 5-HTP into 5-HI in human fecal samples (**Fig 1**), 16S rRNA sequencing data of the fecal samples were analyzed (**Table B in S1 Table**). Of the main differences in the microbial relative abundance among the 3 classes of Converters was the Bifidobacterium genus, which increased from 0.6% in the High Converters, to 9.5% in the Intermediate Converters, to 25.4% in the Non-Converters (**Figs 3A and S4A**). These results were further confirmed by principal component analysis (PCA) and redundancy analysis (RDA) (**S4B and S4C Fig and Table C in S1 Table**). The PCA showed clear separation of the High-Converters from Intermediate-Converters and Non-Converters. Similarly, RDA showed significant influence of 5-HI levels, being the explanatory variable (*p*-value = 0.017), which was positively related to the Bifidobacterium in the Intermediate and Non-Converters group. Surprisingly, the relative abundance of tnaA-encoding bacteria did not significantly change among the 3 tested groups neither when Pearson R correlations were performed to correlate 5-HI levels with tnaA-encoding bacteria, as a group, or the individual respective taxa (**Table D in S1 Table**), implying that it is not the presence of tnaA-encoding genera but rather the presence of Bifidobacterium that derives the observed differences in 5-HI production. To further confirm that the presence of high abundance of Bifidobacterium in the samples of Non-Converters resulted in inhibiting the conversion of 5-HTP by TnaA enzyme, we incubated *F*. *nucleatum* subsp. *animalis*, which fully converted 5-HTP to 5-HI (**Fig 2E**) anaerobically with either 10% or 1% of *Bifidobacterium breve* and 5-HTP conversion to 5-HI was analyzed using HPLC-ED/UV. Remarkably, 5-HI production was altered when *F*. *nucleatum* subsp. *animalis* was incubated with 100 μM 5-HTP in the presence of 10% and 1% of *B*. *breve* but not in the absence of *B*. *breve* (**Fig 3B–3D**). We expected *B*. *breve* to change the pH of the growth media via its production of lactic acid or acetic acid [36], which might have effect on the TnaA activity that is strongly dependent on the pH conditions [37]. Indeed, the pH measurements revealed that co-culturing of *B*. *breve* with *F*. *nucleatum* subsp. *animalis* decreased the pH of the culture from 7.3 before the start of the incubation to pH = 5.34, 6.02, and 7.14 after 24 h of incubation with 10%, 1%, and 0% *B*. *breve*, respectively (**Fig 3B–3D**). Taken together, the results indicate an inhibitory effect of Bifidobacteria on the conversion of 5-HTP into 5-HI by *F*. *nucleatum* subsp. *animalis*.

To investigate what the effect of pH change in the extracellular environment on the *F*. *nucleatum* subsp. *animalis* cells is, *F*. *nucleatum* subsp. *animalis* was cultured in Enriched Beef Broth culture media at different pH levels ranging from pH 3 to 7. Intriguingly, only bacteria cultured in Enriched Beef Broth (pH 7) had normal growth (**S4D Fig**). These data clearly show that the reduction in the pH results in a complete inhibition of the production of 5-HI via its effect on the bacterial growth.

It has been previously shown that the tnaA activity is pH dependent [37]. To further confirm that altered pH levels intracellularly is also responsible for inhibition of TnaA activity, cell lysates of *F*. *nucleatum* subsp. *animalis* and *B*. *thetaiotamicron* were incubated with 100 μM 5-HTP at 37 °C at different pH levels. Samples were collected after 48 h and were analyzed using HPLC-ED/UV. Indeed, production of 5-HI decreased from 88 ± 0.4 and 76 ± 0.7 μM at pH = 7 and 6, respectively, to 2 ± 0.2 μM at pH = 5, to no production of 5-HI at pH = 4 and 3 in *F*. *nucleatum* subsp. *animalis* (**S4E Fig**) and from 28 ± 0.3 and 0.2 ± 0.2 μM at pH = 7 and 6, respectively, to no production of 5-HI at pH = 5, 4, and 3 in *B*. *thetaiotamicron* (**S4F Fig**), confirming that TnaA activity is inhibited at low pH [37].

### 5-hydroxyindole accelerates GI motility in vivo

**Fig 4:**
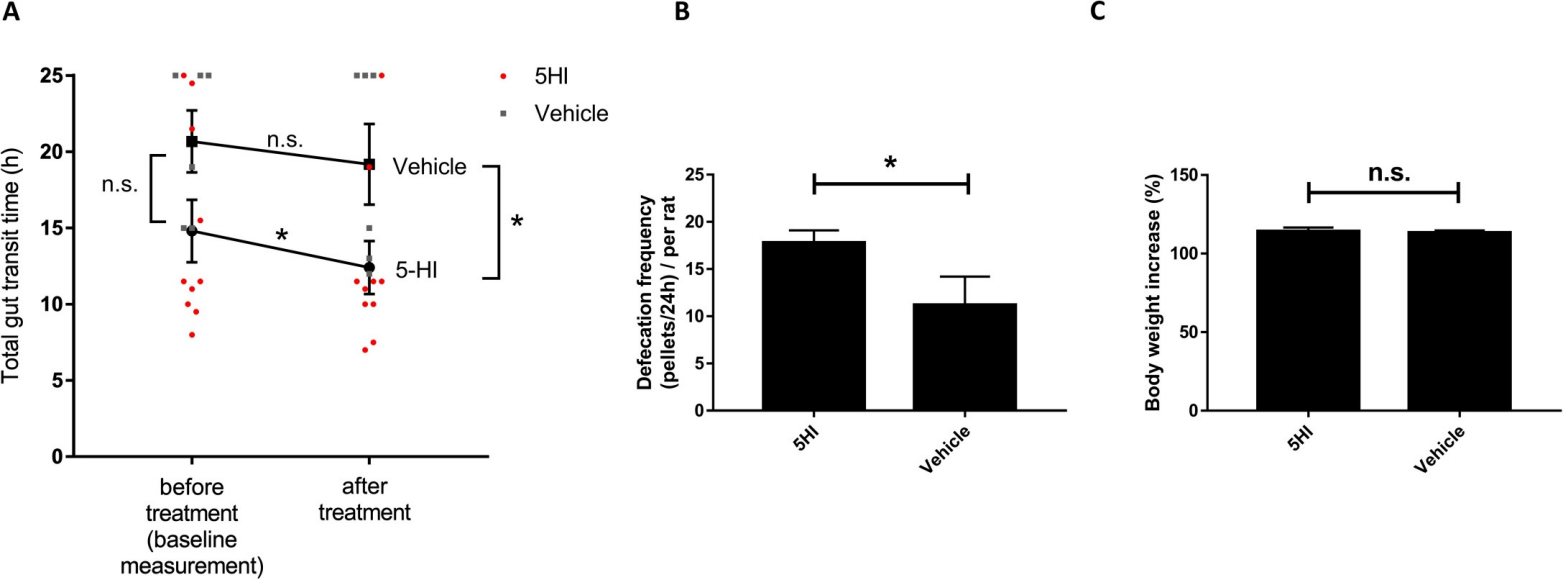
5-HI stimulates GI motility in vivo. **(A)** Graph shows effect of 5-HI on TGTT in WTG rats. First, baseline measurement (before treatment) of the TGTT in all WTG rats was performed. After treatment, TGTT was significantly decreased in WTG rats treated with 30 mg/kg of 5-HI (black circles, *n* = 10; red circles show single measurement points in each rat before and after treatment) but not in the vehicle group treated with 10% sucrose (black squares, *n* = 6; gray squares show single measurement points in each rat before and after treatment). Data were analyzed using the RM 2-way ANOVA followed by Fisher LSD test (*p < 0.05) (before and after treatment) and 2-tailed unpaired *t* test (*p < 0.05) was used for analysis of data from 5-HI-treated group and vehicle group after treatment. Error bars represent SEM. (**B)** Increased defecation frequency per 24 h per rat in rats treated with 30 mg/kg of 5-HI compared to the vehicle group (10% sucrose). Data were analyzed using the 2-tailed unpaired *t* test (*p < 0.05). Error bars represent SEM. (**C)** Bar graph showing no difference between body weight increase in WTG rats treated with 30 mg/kg of 5-HI compared to the vehicle group. Data were analyzed using the 2-tailed unpaired *t* test (n.s. > 0.05). Error bars represent SEM. The raw data used for quantification of **A, B,** and **C** can be found in S3 Data. 5-HI, 5-hydroxyindole; GI, gastrointestinal; LSD, least significant difference; n.s., not significant; RM, repeated measures; TGTT, total gut transit time; WTG, wild-type Groningen.

Being the bacterial metabolite of 5-HTP that has been recently shown to accelerate the gut motility [20], we sought to test 5-HI for its ability to also affect gut motility in vivo. To this end, we orally administered 30 mg/kg of 5-HI to wild-type Groningen (WTG) adult male rats (*n* = 10). First, baseline measurements of total gut transit time (TGTT) were performed in all rats. Baseline TGTT measurements showed a natural variation among the WTG rats, similar to what exists among humans. Subsequently, the rats were randomly appointed to either vehicle (10% sucrose) group or 5-HI-treated group, and TGTT was measured in both groups. Remarkably, the 5-HI-treated group had significantly decreased TGTT compared to the baseline measurements before treatment (**Fig 4A**). Moreover, the defecation frequency was significantly increased in the 5-HI-treated group compared to the vehicle group (**Fig 4B**), without affecting food intake or changes in body weight (**Fig 4C**). Together, the in vivo data suggest that 5-HI stimulates the GI motility in rats. However, the underlying mechanisms by which 5-HI exerts its effect remain obscure.

### 5-hydroxyindole mediates its effect on colonic contractility via 2 distinct mechanisms

**Fig 5:**
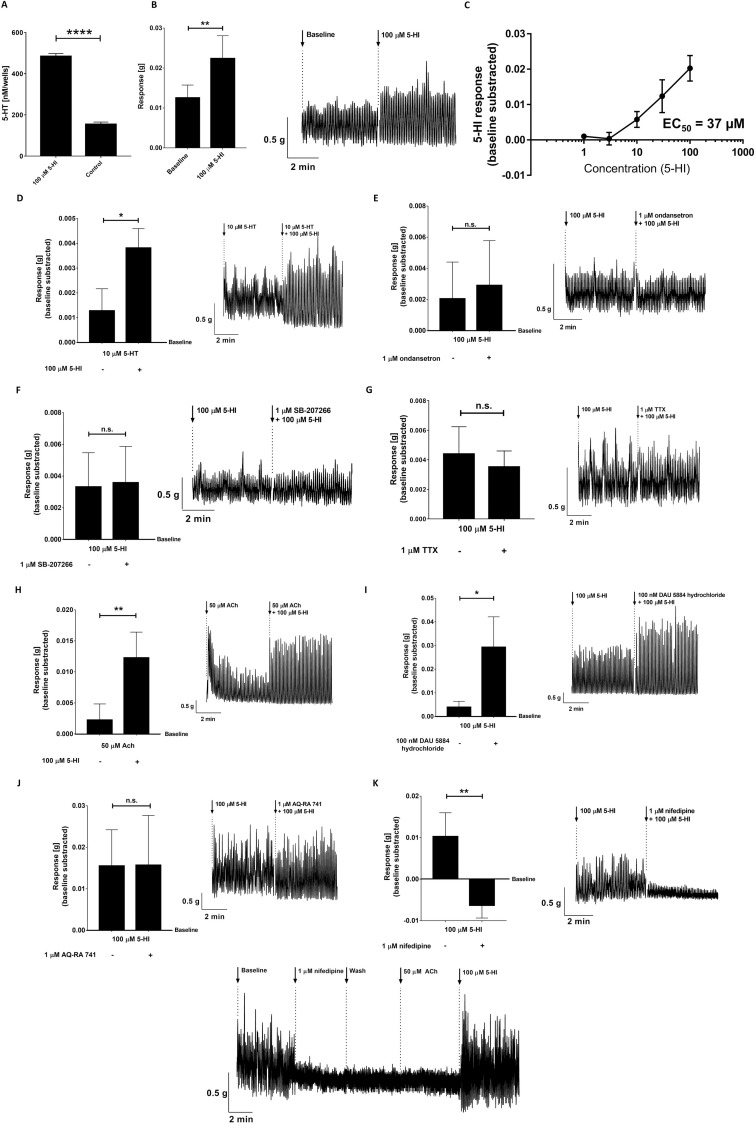
5-HI and its effect on colonic contractility. **(A)** Bar graph represents a significantly increased 5-HT release from RIN14B cells after stimulation with 100 μM of 5-HI. Data were analyzed using the 2-tailed unpaired *t* test (****p < 0.0001). Error bars represent SEM. (**B)** Bar graph and illustrative recording in the rat colon represents the enhanced response of the 5-HI on the gut contractility. (**C)** The dose response curve and EC_50_ value of 5-HI in the rat colonic tissue. **(D–G)** Bar graphs and illustrative recordings in the rat colon represent the enhanced response of the 5-HT-induced contractility by (**D)** 100 μM 5-HI, which was not inhibited after adding (**E)** 1 μM ondansetron, (**F)** 1 μM SB-207266, or (**G)** 1 μM TTX. (**H–J)** Bar graphs and illustrative recordings in the rat colon represent enhanced response of the ACh-induced contractility by (**H)** 100 μM 5-HI, which was significantly increased after adding (**I)** 100 nM DAU 5884 hydrochloride and was not inhibited after the addition of (**J)** 1 μM AQ-RA 741. (**K)** Addition of 1 μM nifedipine significantly inhibited 5-HI-induced response. **(L)** Illustrative recording in the rat colon represents enhanced response by 100 μM 5-HI after colonic tissue contractile apparatus is altered by nifedipine. Data represent 3–9 biological replicates. Data were analyzed using the Wilcoxon matched-pairs (before/after) signed rank test (*p < 0.05; **p < 0.01). Error bars represent SEM. Quantitative analysis of the organ bath data is described in Materials and method section. The raw data used for quantification of **A–K** can be found in S4 Data. 5-HI, 5-hydroxyindole; ACh, acetylcholine; n.s., not significant; TTX, tetrodotoxin.

To decipher the mechanisms underlying the stimulatory effect of 5-HI on gut motility observed in vivo (**Fig 4**), we first evaluated the effect of 5-HI on 5-HT release in RIN14B cells, a model cell line of enterochromaffin cells [38]. When RIN14B cells were stimulated with 100 μM 5-HI (concentration of 5-HI employed was based on the 5-HI levels detected in the fecal samples from healthy participants (**Fig 1A**), 5-HT release was significantly increased (484 ± 14 nM) compared to the control (154 ± 12 nM; **Fig 5A**). 5-HT released from enterochromaffin cells modulates the intestinal motility via activation of 5-HT_3_ and 5-HT_4_ receptors on afferent nerve terminals from the ENS [13].

In parallel, we observed the ability of 5-HI to translocate through the intestinal tissue **(S5A Fig)**, as well as to cross the first pass metabolism as indicated by its detection in urine samples of rats after administration of 30 mg/kg 5-HI (3.5 ± 1.3 μM). These data suggest that 5-HI may reach the GI motility control system and induce GI contractions via a second mechanism besides its stimulation of 5-HT production. Thus, we employed an ex vivo organ bath system [39], where dissected proximal colonic tissues with intact mucosa from untreated WTG rats were cut to approximately 3 mm rings and suspended in an organ bath as described before [40]. A pressure transducer and data-acquisition software were employed to display the measurement of tension generated by the smooth muscle of intestinal walls. We focused on the colon since we observed that the small intestinal transit lasted only approximately 15 min in average, while the large intestinal transit ranged from 10 h to 24 h (**Fig 4A)**.

When 100 μM 5-HI was applied to the colonic tissue, a significant increase in the contractility was observed compared to the baseline (quantification of the data is described in Materials and methods section; **Fig 5B**). Next, we performed a dose–response curve of 5-HI, and EC_50_ was determined to be 37 μM (**Fig 5C**), confirming that 5-HI will produce its maximum effect at the selected dose.

To further investigate whether 5-HI can augment the stimulatory effect of 5-HT on colonic contractility, 100 μM 5-HI was applied sequentially with 10 μM 5-HT to the colonic tissue in the organ bath system. 5-HT concentration was based on previous reports showing levels of 5-HT in the mucosa from variety of animal models and human specimens [21]. Interestingly, when applied after 10 min of the 5-HT exposure (this timing is used in all the following organ bath experiments), the addition of 5-HI elicited significantly higher amplitude in the tissue contractility compared to the 5-HT-induced response (**Fig 5D)**.

To test whether the augmented amplitude enhancement of the 5-HT-induced response upon addition of 5-HI was due to the action of 5-HI on the 5-HT_3_ or 5-HT_4_ receptors, the main 5-HT receptors involved in the gut motility [41], the 5-HT_3_ antagonist ondansetron (1 μM) or the 5-HT_4_ antagonist SB-207266 (1 μM) were added to the colonic tissue to abolish the 5-HT-induced response [42,43]. Addition of either ondansetron or SB-207266 sequentially with 5-HT or 5-HI, respectively, resulted in an inhibition of 5-HT- but not the 5-HI-induced response (**Figs 5E and 5F and S5B and S5C**), suggesting that 5-HI does not act via 5HT_3_ or 5-HT_4_ receptors to stimulate the colonic contractions.

To further investigate whether 5-HI facilitates its action via receptors on either enteric nerves or smooth muscle, 1 μM tetrodotoxin (TTX) [44], a neurotoxin that prevents axonal transmission by blocking sodium ion transport [45], or 1 μM hexamethonium, a nicotinic cholinergic antagonist often referred to as the prototypical ganglionic blocker [46], was added to the colonic tissue. Addition of both TTX and hexamethonium had an inhibitory effect on the response of 5-HT (**S5D and S5E Fig**), but did not alter the response of 5-HI (**Figs 5G and S5F**), indicating that the receptors by which 5-HI exerts its effect on gut contractility are located on the smooth muscle, as is the case for the ACh-induced response [45].

Next, the neurotransmitter ACh (50 μM) was applied to the tissue to induce a maximum increase in the intestinal smooth muscle tone [47], which has been long recognized as a key component in gut motility [48]. Intriguingly, addition of 100 μM 5-HI significantly intensified the amplitude of the ACh-induced contractility in the colonic tissue (**Fig 5H**). The ACh response is mainly exerted through muscarinic ACh receptor subtypes 2 (M2R) and 3 (M3R) located on the smooth muscle cells in the GI tract [6]. To test whether 5-HI exhibits its action via either M2R or M3R, AQ-RA 741 (1 μM) or DAU 5884 hydrochloride (100 nM), selective antagonists of the M2R or M3R, respectively [49,50], were applied sequentially with 5-HI or ACh to the tissue. As expected, the ACh-induced response was inhibited by the addition of either the M2R or M3R antagonist (**S5G and S5H Fig**). In contrast, there was a significant increase in the 5-HI-induced response when M3R was inhibited (**Fig 5I**), and the addition of M2R antagonist did not inhibit the 5-HI-induced response (**Fig 5J**), suggesting that 5-HI exerts its excitatory effect on the colonic smooth muscles through other receptors.

**Fig 6:**
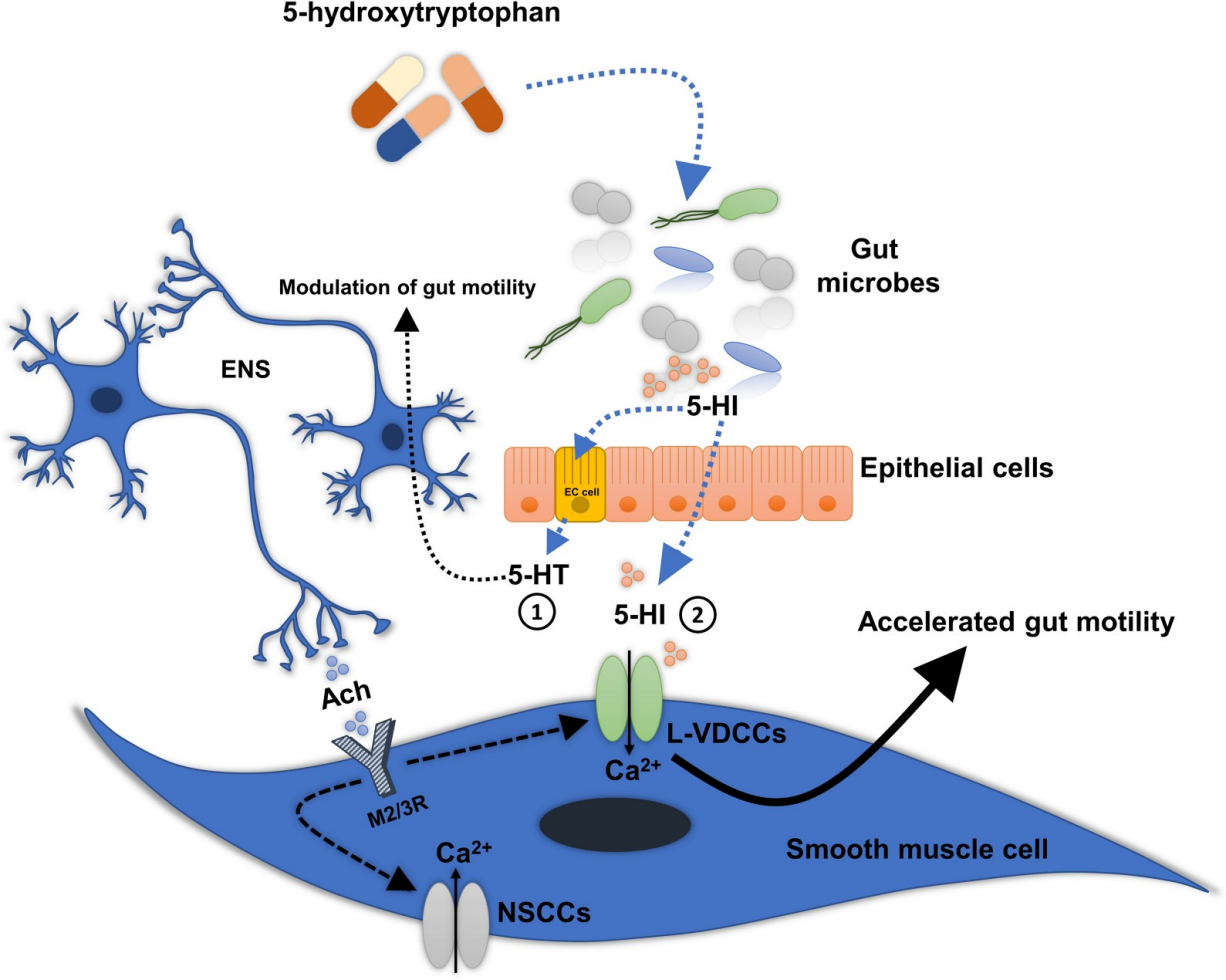
Proposed model for the mechanism by which 5-HI accelerates intestinal motility. Following bacterial conversion of 5-HTP to 5-HI, serotonin (5-HT) is released from EC cells to possibly modulate the intestinal motility via activation of 5-HT_3_ and 5-HT_4_ receptors on afferent nerve terminals from the ENS. In parallel, 5-HI translocates through the gut tissue, where it reaches the smooth muscle cells and accelerates colonic motility directly via activation of L-VDCCs. 5-HI, 5-hydroxyindole; 5-HTP, 5-hydroxytryptophan; EC, enterochromaffin; ENS, enteric nervous system; L-VDCCs, L-type voltage-dependent Ca^2+^ channels; NSSCs, nonselective Ca^2+^ channels.

Typically, the muscle contractions are caused by Ach-induced opening of a variety of cationic channels (e.g., nonselective Ca^2+^ channels, specifically the interconnected transient receptor potential channel 4 (TRPC4) and 6 (TRPC6) [51] and L-type voltage-dependent Ca^2+^ channels (L-VDCCs) [44]) in the smooth muscle cells of the GI tract, thereby producing Ca^2+^ influx. To test whether nonselective Ca^2+^ channels or L-VDCCs are involved in the 5-HI-induced gut contractility, ACh was applied sequentially with 5-HI followed by the addition of selective antagonists of TRPC4 and TRPC6, ML 204 (1 μM) and SAR 7334 (1 μM), respectively [51]. Only the ACh-induced response, but not the 5-HI-induced response, was inhibited (**S5I and S5J Fig**), respectively. Next, 5-HI was applied sequentially with 1 μM nifedipine [52], a nonselective antagonist of L-VDCCs. Remarkably, the 5-HI-induced response was completely abolished with the addition of nifedipine (**Fig 5K**), suggesting that 5-HI elicits its response via L-VDCCs. Moreover, nifedipine is thought to block the contractile apparatus of the smooth muscle [53]. Therefore, to validate an independent contractile agent in the presence of nifedipine, 100 μM of ATP, which causes contraction mainly by the release of Ca^2+^ from intracellular Ca^2+^ stores, was added to the colonic tissue. Indeed, colonic contractility induced by ATP was only partially inhibited by nifedipine compared to when ATP was not present (**S5K Fig**). Finally, to recover the spontaneous contractions of the colonic tissue after the nifedipine treatment (nifedipine was washed away from the tissue in the organ bath), only 5-HI but not ACh was able to restore the tissue to the baseline contractions before nifedipine treatment (**Fig 5L**) showing 5-HI to be a potent stimulant of the colonic contractility. Collectively, these results show that 5-HI mediates its effect on gut contractility via its induction of L-VDCCs on the smooth muscle cells of the GI tract, which, in turn, increases intracellular Ca^2+^ (**Fig 6**) and possibly via its induction of 5-HT production from the enterochromaffin cells (**Fig 5A**).

## Discussion

By combining a suite of in vitro biochemical, culture-based, and organ bath assays, microbiota sequencing, in vivo animal experiments, and human intervention study, we have identified the potency of 5-HI, a product of gut microbial conversion of the dietary supplement and antidepressant 5-HTP, to accelerate GI motility directly via activation of L-type calcium channels located on the colonic smooth muscle cells and possibly through its induction of 5-HT production from enterochromaffin cells. Of note, 5-HI is often wrongly associated with 5-hydroxyoxindole [35,54], which is an oxindole with a hydroxyl group at position 5 of the indole ring, and is detected in blood, plasma, and the brain [55].

Our primary outcome is that a wide variety of small and large intestinal bacteria possessing TnaA activity, typically converting tryptophan to indole, can efficiently convert 5-HTP into 5-HI in the intestinal lumen, with variability in the conversion levels (**Figs 1A, 2E and 2F and S2C**). The differences in the conservation level detected at the TnaA active sites [32] among the 15 closest orthologues of *E*. *coli*, *B*. *thetaiotamicron*, and *F*. *nucleatum* (**S3 Fig**) may explain the observed variation in the production of 5-HI among the tested bacterial strains. Moreover, the variation in the production of 5-HI in the human fecal samples was associated with high relative abundance of Bifidobacteria in the human fecal samples (**Figs 3 and S4A**), which is consistent with previous data showing that Bifidobacterium spp. isolated from healthy individuals inhibited the TnaA activity of *E*. *coli* or total human intestinal microbiota [56]. Similarly, indole levels are reported to be decreased in the infant fecal samples, which are dominated by Bifidobacteria compared to fecal samples from adults [57]. The inhibitory effect of Bifidobacteria observed in this study is due to the ability of Bifidobacteria to decrease the pH of the samples (**Fig 3B–3D**), which could affect the growth of other tnaA-encoding bacteria **(S4D Fig)** or the TnaA activity **(S4E Fig)** which was previously shown to be pH dependent [37].

Metabolites with similar chemical structures, such as indole and indole derivatives, have large effects on host [58–61], thus it was expected that 5-HI will also influence host physiology [62–64]. Deciphering the mechanism of 5-HI stimulatory effect on gut motility observed in WTG rats (**Fig 4**), we first demonstrated that 5-HI significantly increased 5-HT release from a model of enterochromaffin cells, RIN14B (**Fig 5A**). Notably, gut microbiota-derived products, such as short-chain fatty acids, secondary bile acid (deoxycholate), and saturated long-chain fatty acids, have previously been shown to directly modulate enterochromaffin cell function and alter GI motility [3,4,65,66]. Our result that 5-HI stimulates 5-HT release from a model of enterochromaffin cells, RIN14B cell line, indicates that gut microbiota-derived metabolites affect 5-HT release thus maybe involved in modulating the host gut motility [3,4,9,67].

However, the fact that 5-HI also strongly stimulates altered contractions in the rat colonic tissue after nifedipine treatment (nifedipine is washed away from the tissue in the organ bath; **Fig 5L**), possibly via a rise in Ca^2+^ influx, grants 5-HI another mechanism how to alter gut motility. Our data showed that 5-HI is able to increase the stimulatory effect of 5-HT and that this effect is not via stimulation of 5-HT_3_ and 5-HT_4_ receptors or its action via neuronal action potentials (**Figs 5E–5G and S5F**). Kooyman and colleagues have shown that 5-HI, when used as a chemical to mimic the aromatic moiety of 5-HT, could modulate 5-HT-induced desensitization of the 5-HT_3_ receptor-mediated inward current in murine neuroblastoma cells [68]. The observed 5-HI effect occurred via a competitive or noncompetitive interaction at low (10 mM) or high (10 to 50 mM) concentrations, respectively. These concentrations are 100 to 500 times higher than the concentration employed in the present study (100 μM), which was based on the 5-HI levels detected in the fecal samples from healthy participants (**Fig 1A**) and the EC_50_ value **(Fig 5C)**. The differences in concentrations of 5-HI used by Kooyman and colleagues and in the present study may explain the discrepancy in the results. Moreover, the biophysical properties of the 5-HT_3_ receptors expressed in the murine neuroblastoma cells used in the study of Kooyman and colleagues could widely differ from those expressed in the rat colonic tissue as previously described [69]. Our findings showing that 5-HI does not have stimulatory effect on 5-HT_3_ receptors is further supported by the application of ondansetron, a selective antagonist of 5-HT_3_ receptors, TTX and hexamethonium, which both blocks neuronal transmission (**Figs 5E and S5D and S5F**).

The increased 5-HI-induced smooth muscle contraction upon inactivation of the M3R (**Fig 5I)** is most likely because when activated, M3R, the main receptor mediating contractions in the smooth muscle [70], leads to inactivation of VDCCs followed by lower Ca^2+^ discharge [7,71]. Through its direct activation of the L-VDCCs (**Fig 5K**), 5-HI was most likely able to restore the entry of Ca^2+^ when M3R was blocked. This is also supported by 5-HT release experiment (**Fig 5A**), where enterochromaffin cells are endowed with L-VDCCs and 5-HT release largely depends on Ca^2+^ influx into the cells [72]. L-VDCCs are also known as voltage-gated ion channels and are densely expressed in the smooth muscle cells of the GI tract [44,73]. Specifically, a subtype of L-VDCCs, L-type Ca_v_1.2 Ca^2+^ channel, was described as essential for precise functioning of gut contractility in intestinal smooth muscle [44]. Moreover, altered expression or function of L-VDCCs in the gut was shown to cause GI motility disorders, including constipation [53,74,75]. Indeed, L-VDCCs agonists may serve to restore intracellular Ca^2+^-dependent motility to the constipated colon by activating the Ca^2+^ channels [75]. This claim is further strengthened by our results where the oral administration of 5-HI reduced TGTT in WTG rats (**Fig 4A**). However, a main limitation in the current study is the lack of motility measurements in human volunteers, who administered 5-HTP to address the impact of 5-HI on intestinal motility.

Our newfound understanding of the formation of 5-HI by a wide range of TnaA-harboring bacteria will guide how therapeutic communities of microorganisms can be designed in which the production of 5-HI can be genetically specified. However, despite the fact that 5-HI maybe present at micromolar concentration in the gut of individuals who administer 5-HTP, its levels can vary greatly among individuals (from 0.1 to 13.2 μM) depending on the environmental conditions (**Fig 3B–3D**). Against this backdrop, delivering 5-HI microbial metabolite as a targeted drug into the human gut to stimulate gut contractility may represent a significant advance towards precision treatment of diseases, where constipation is a risk factor.

## Materials and methods

### Study participants and fecal specimen collection

The study protocol was evaluated by the ethical committee of University of Groningen Medical center. Eighteen volunteers (8 male and 10 female) between the ages 20 and 40 were recruited. Participants were excluded if they had used antibiotics, diarrhea inhibitors, laxatives, proton pump inhibitors, or had any GI discomfort within the last 3 weeks. The study coordinator met with each eligible participant to review the consent and study details. All participants signed the confidentiality and consent form.

A plastic collection container, relief container, and gloves were provided to each subject for fecal collection. Participants collected the feces and delivered it immediately to the study coordinator who delivered it to the laboratory for processing. One part of the fecal specimen was homogenized in Liquid Amies medium containing 20% glycerol (**Table E in S1 Table**), snap-frozen, and stored in −80 °C. The second part was only snap-frozen without any additives in liquid nitrogen and stored in −80 °C.

### Fecal specimen incubation

Fecal samples were suspended in an Enriched Beef Broth (EBB) based on SHIME medium [76] (**Table F in S1 Table**) (5% w/v). Samples were incubated anaerobically (10% H_2_, 10% CO_2_, 80% N_2_) in a Don Whitley Scientific DG250 Workstation (LA Biosystems, Waalwijk, the Netherlands) at 37 °C overnight prior to the addition of 100 μM 5-hydroxy-L-tryptophan (5-HTP; H9772, Sigma). Subsequently, samples containing 5-HTP were further incubated anaerobically at 37 °C for 6 and 24 h, prior to HPLC-UV analysis.

### HPLC-ED/UV analysis and sample preparation

A volume of 1 mL of ice-cold methanol was added to 0.25 mL bacterial cell suspensions and fecal samples suspensions and stored at −20 °C until further use. Cell and protein precipitates were removed by centrifugation at 20,000 × g for 10 min at 4 °C. Supernatant was transferred to a new tube, and the methanol fraction was evaporated in a Savant speed-vacuum dryer (SPD131, Fisher Scientific, Landsmeer, the Netherlands) at 60 °C for 2 h. The aqueous fraction was reconstituted to 1 mL with MilliQ-filtered water. Samples were filtered and injected into the HPLC system. For *E*. *coli* BW25113 tnaA mutant experiment and Fusobacterium strains screening experiment, HPLC with electrochemical detection was used (Jasco AS2059 plus autosampler, Jasco Benelux, Utrecht, the Netherlands; Knauer K-1001 pump, Separations, H. I. Ambacht, the Netherlands; Dionex ED40 electrochemical detector, Dionex, Sunnyvale, USA, with a glassy carbon working electrode (DC amperometry at 1.0 V, with Ag/AgCl as reference electrode), for every other experiment HPLC with UV detection (280 nm) was used (Waters 2695 Alliance Separations module, Milford, Massachusetts, USA; UV6000LP Detector, Thermo Fisher Scientific, Waltham, Massachusetts, USA). Samples were analyzed on a C18 column (Kinetex 5 μm, C18 100 Å, 250 × 4.6 mm, Phenomenex, Utrecht, the Netherlands) using a gradient of water/methanol with 0.1% formic acid (0 to 10 min, 95% to 80% H_2_O; 10 to 20 min, 80% to 5% H_2_O; 20 to 23 min, 5% H_2_O; 23 to 31 min, 95% H_2_O). Data recording and analysis were performed using Chromeleon software (version 6.8 SR13).

### LC-MS analysis

LC-MS analysis was performed using an Accella1250 HPLC system coupled with the benchtop ESI-MS Orbitrap Exactive (Thermo Fisher Scientific, Waltham, Massachusetts, USA) on positive ion mode. Samples were analyzed on a C18 column (Shim Pack Shimadzu XR-ODS, 2.2 μ 3 × 75 mm, Shimadzu Corporation, Kyoto, Japan) or on XB-C18 column (Kinetex 2.6 μm, XB-C18, 100 Å, 150 x 1.0 mm, Phenomenex, Utrecht, the Netherlands) using a gradient of water/acetonitrile with 0.1% formic acid (0 to 5 min, 98% to 90% H_2_O; 5 to 10 min, 90 to 5% H_2_O; 10 to 13 min, 5% H_2_O; 13 to 14 min, 98% H_2_O). Data analysis was performed using Qual Browser Thermo Xcalibur software (version 2.2 SP1.48).

### Oral 5-HTP administration (human study)

The study protocol was evaluated by the ethical committee of University of Groningen Medical center. Five out of the 18 tested participants who participated in the fecal samples donation were randomly selected in a blind manner and asked to orally administer 5-HTP tablets (Swanson Health Products, Fargo, North Dakota, USA) (50 mg 5-HTP per tablet) daily for 1 week, as recommended by the manufacturer. The study coordinator met with each participant to review the consent and study details. All participants signed the confidentiality and consent form. Dietary intake of L-tryptophan was not regulated. Volunteers collected fecal samples on Day 0 (control, no 5-HTP ingested), Day 1 (first ingestion of 5-HTP tablet), Day 3, and Day 8 (1 day after last 5-HTP was ingested), respectively. A plastic collection container, relief container, and 5-HTP tablets were provided to each participant for fecal collection on specified days. Participants collected the feces and delivered it immediately to the study coordinator who delivered it to the laboratory for fecal samples extraction of metabolites. Samples collected on Day 0 were used also for determination of basal levels of 5-HTP conversion (procedure described in fecal specimen incubation). After blind analysis, 4 volunteers turned out to belong to the High Converters group, and 1 volunteer belonged to the Intermediate Converters group. The rest of the samples were stored in −80 °C.

### Fecal samples extraction of metabolites

After fecal specimen collection, part of the sample was weighed, and MilliQ-filtered water was added (1:5 w/v). Solution was homogenized thoroughly. Next, 250 μL of this solution was transferred to 1 mL of 100% ice-cold methanol. Samples were stored at −20 °C until analysis by HPLC-UV.

### DNA extraction and 16S rRNA gene profiling

To determine the possible cause of variation in the bacterial conversion of 5-HTP into 5-HI in human fecal samples, we analyzed 16s rRNA sequencing data available for 9 fecal samples (BioProject: PRJNA683576) of the participants that were employed in this study. DNA extraction was performed using the Quick-DNA Fecal/Soil Microbe Miniprep Kit (Zymo Research) according to manufacturer’s instructions. Illumina 16S rRNA gene amplicon libraries were generated and sequenced at BaseClear (Leiden, the Netherlands). In short, barcoded amplicons from the V3-V4 region of 16S rRNA genes were generated using a 2-step PCR. A total of 10 to 25 ng genomic (g)DNA was used as template for the first PCR with a total volume of 50 μL using the 341F (5′-CCTACGGGNGGCWGCAG-3′) and the 785R (5′-GACTACHVGGGTATCTAATCC-3′) primers appended with Illumina adaptor sequences. PCR products were purified, and the size of the PCR products were checked on Fragment analyzer (Advanced Analytical) and quantified by fluorometric analysis. Purified PCR products were used for the second PCR in combination with sample-specific barcoded primers (Nextera XT index kit, Illumina). Subsequently, PCR products were purified, checked on a Fragment analyzer (Advanced Analytical), and quantified, followed by multiplexing, clustering, and sequencing on an Illumina MiSeq with the paired-end (2x) 300 bp protocol and indexing. The sequencing run was analyzed with the Illumina CASAVA pipeline (v1.8.3) with demultiplexing based on sample-specific barcodes. The raw sequencing data produced were processed removing the sequence reads of too low quality (only “passing filter” reads were selected) and discarding reads containing adaptor sequences or PhiX control with an in-house filtering protocol. A quality assessment on the remaining reads was performed using the FASTQC quality control tool version 0.10.0. The Illumina paired reads were merged into single reads (so-called pseudoreads) through sequence overlap, after removal of the forward and reverse primers. Chimeric pseudoreads were removed using USEARCH 9.240, and the remaining reads were aligned to the RDP 16S rRNA gene database (RDP Release version 11). Based on the alignment scores of the pseudoreads, the taxonomic depth of the lineage is based on the identity threshold of the rank; Species 99%, Genus 97%, Family 95%, Order 90%, Class 85%, Phylum 80%. Shannon entropy of counts, a metric of microbiota diversity, was calculated using USEARCH 9.2 with OTU clustering with a sequencing identity threshold of 97% after subsampling from the entire set, to account for different sampling depths.

### Principal component and redundancy analysis

Microbiome analysis was done with the statistics software R (R version 3.6.1, https://www.R-project.org/). Raw OTU counts were first normalized by cumulative sum scaling (CSS) using methods from the *metagenomeSeq* package [77], and PCA was carried out leveraging the *ordinatate* method from *phyloseq* [78]. RDA constrained to 5-HI levels as a single explanatory variable was performed in the same manner, while also testing for significance via an anova-like permutation for the significance of the constraint. Visualization was done using *ggpubr* (https://cran.r-project.org/web/packages/ggpubr/index.html).

### Bacteria

*E*. *coli* DH5a and BL21 were routinely grown aerobically in Luria-Broth (LB) at 37 °C degrees with continuous agitation. Other strains listed in **Table G in S1 Table** were grown either aerobically at 37 °C with continuous agitation or anaerobically at 37 °C in an EBB unless otherwise noted. Bacteria were inoculated from −80 °C stocks and grown overnight. Before the experiment, cultures were diluted 1:100 in fresh medium from overnight cultures. 5-HTP was supplemented during the lag or stationary phase depending on the experiment. Growth was followed by measuring the optical density (OD) at 600 nm in a spectrophotometer (UV1600PC, VWR International, Leuven, Belgium).

*F*. *nucleatum* subsp. *animalis* DSM 19679 and *B*. *breve* DSM 20213 were grown anaerobically at 37 °C in an EBB. *F*. *nucleatum* subsp. *animalis* and *B*. *breve* were first restreaked on Fastidious Anaerobe Agar supplemented with 5% sheep blood or on MRS agar, respectively, and grown overnight before inoculation to liquid EBB culture. Before the experiment, 10 mL of *B*. *breve* was centrifuged, and cells were washed in fresh 10 mL EBB. From the 100% *B*. *breve* suspension, 10% and 1% of *B*. *breve* suspensions were prepared. To each of the suspensions, 1% of the *F*. *nucleatum* subsp. *animalis* culture was inoculated. Also, *F*. *nucleatum* subsp. *animalis* was inoculated 1:100 to fresh medium, which served as a control. To each of the suspensions, 100 μM 5-HTP or sterile MilliQ-filtered water was supplemented. pH of the cultures was measured, and 0.25 mL of each culture was taken for HPLC-UV analysis at t = 0 h and t = 24 h.

### Cell lysates preparation and assays

Grown overnight cultures of *B*. *thetaiotamicron* VPI-5482 and *F*. *nucleatum* subsp. *animalis* DSM 19679 were diluted 1:100 to a fresh medium (100 mL). Chopped meat medium (CMM; 10 g/L beef extract, 30 g/L casitone, 5 g/L yeast extract, 5 g/L K_2_HPO_4_, 0.1% Tween 80, after autoclavation CMM was supplemented with 1 μg/mL vitamin K3, 0.5 g/L cysteine and 0.005 mg/mL hemin). CMM was used for *B*. *thetaiotamicron* VPI-5482, and for *F*. *nucleatum* subsp. *animalis*, EBB (**Table F in S1 Table**) was used. Next day, 100 mL of grown cultures was harvested, and pellets were washed 3 times in 1 mL 1X PBS. After washing, pellets were resuspended in a combined volume of 2 mL of ice-cold 50 mM phosphate buffer (pH 7.4). Before lysis, 2 μg/mL DNase and 200 μg/mL lysozyme were added to each solution, samples were transferred to screw-caped tubes containing 0.5 g of 0.1 mm zirconia glass beads and kept on ice for 10 min. Samples were homogenized for 35 s with 1-min intervals on ice in a mini bead-beater (Biospec, Bartlesville, USA) for 3 times. After lysis, clear cell lysates were obtained by centrifugation for 20 min at 20,000 × g at 4 °C. Total protein concentration was determined by Bradford assay. Cell lysates assays were performed at 37 °C and with 100 μM 5-HTP. Incubations were performed at 0.2 mg/mL of cell lysates in either 50 mM phosphate buffer (pH 7.4) or citrate buffers (pH 6, 5, 4, and 3). Reactions were initiated by addition of prewarmed cell lysate to prewarmed reaction mixture (100 μM 5-HTP, 100 μM PLP, and either phosphate of citrate buffer). A total of 100 μL samples were collected and quenched in 400 μL ice-cold 100% methanol at 0 and 48 h after initiation. Samples were stored at −20 °C until analysis by HPLC-UV.

### Cloning and heterologous gene expression

TnaA from *E*. *coli* BW25113 (TnaA_*E*.*coli* BW25113_, accession: CP009273) was amplified using Phusion High-Fidelity DNA polymerase and primers listed in **Table H in S1 Table**. Amplified *tnaA* gene was cloned in pET15b, resulting in pBW002 (**Table I in S1 Table**). The plasmid was maintained in *E*. *coli* DH5α, verified by Sanger sequencing and transformed to *E*. *coli* BL21 (DE3). Overnight culture was diluted 1:100 and grown to OD_600_ = approximately 0.8. Protein translation was induced with 1 mM Isopropyl β-D-1-thiogalactopyranoside (IPTG, 11411446001, Roche Diagnostics), and culture was incubated overnight at 18 °C. The cells were directly used for protein isolation. Cell pellets were resuspended in 1/25th of buffer A (300 mM NaCl; 10 mM imidazole; 50 mM NaH_2_PO_4_ (pH 7.4)) containing 0.2 mg/mL lysozyme (105281, Merck) and 2 μg/mL DNase (11284932001, Roche Diagnostics), and incubated for at least 10 min on ice before sonication (10 cycles of 15 s/on with 30 s cooling at 7 μm amplitude) using Soniprep-150 plus (Beun de Ronde, Abcoude, the Netherlands). Cell debris were removed by centrifugation at 20,000 × g for 20 min at 4 °C. The 6 x his-tagged protein was purified using a nickel-nitrilotriacetic acid (Ni-NTA) agarose matrix (30250, Qiagen). Cell-free extracts were loaded on 0.5 mL Ni-NTA matrixes and incubated on a roller shaker for 2 h at 4 °C. The Ni-NTA matrix was washed 3 times with 1.5 mL buffer B (300 mM NaCl; 20 mM imidazole; 50 mM NaH_2_PO_4_ (pH 7.4)) before elution with buffer C (300 mM NaCl; 250 mM imidazole; 50mM NaH_2_PO_4_ (pH 7.4)). Imidazole was removed from purified protein fraction using Amicon Ultra centrifugal filters (UFC505024, Merck) and washed 3 times and reconstituted in buffer D (50 mM K_2_HPO_4_, 50 mM KH_2_PO_4_ (pH 7.4)). Protein concentration was measured spectrophotometrically (Nanodrop 2000, Isogen, De Meern, the Netherlands) using predicted extinction coefficient and molecular weight from Protein Molecular Weight tool (http://www.bioinformatics.org/sms/prot_mw.html).

### Enzyme kinetics

L-tryptophan and 5-HTP degradation by purified TnaA from *E*. *coli* BW25113 was tested by measuring indole or 5-HI formation, respectively, as previously described [34]. Reactions were performed in biological triplicates using L-tryptophan substrate ranges from 0 to 14.58 mM and 5-HTP substrate ranges from 0 to 12 mM. Enzyme kinetics were performed in 50 mM potassium phosphate buffer containing 0.1 mM PLP (pyridoxal-5-phosphate, P9255, Sigma) and 20 or 200 nM enzyme at pH 7.4. The reaction mixture was prewarmed for 5 min at 37 °C and was initiated by adding an enzyme solution and was terminated afterwards by the addition of 100 μL of 1 M HCl, following the addition of 100 μL of Kovac’s reagent for indoles (60983, Sigma, the Netherlands). The supernatant was examined spectrophotometrically at 540 nm. The amounts of indole and 5-HI formed in each reaction were calculated from a standard curve. Michaelis–Menten kinetic curves were fitted using GraphPad Prism 7.

### Bioinformatics

TnaA amino acid sequences from *E*. *coli* BW25113, *B*. *thetaiotamicron* VPI-5482, and *F*. *nucleatum* subsp. *animalis* ATCC 51191 were retrieved from the NCBI database (accession numbers: AIN34033.1, AAO76599.1, EGQ80312.1) and a BLAST [79] search was locally carried out against the translated HMP Reference Genome (HMRGD) sequence database, which was subsetted to contain only GI tract as body site. Blastp was run in default configuration, and hits with less than 90% query cover or less than 30% identity were discarded to still stay in the safe space for homology modelling. From the resulting sequences, orthologues were inferred by running OrthoFinder [80] with the -M option set to “msa.” The 15 closest (with highest identity) orthologues to each of the initial seed sequences were extracted, and a multiple sequence alignment was performed using MUSCLE [81] in default mode and visualized via JalView [82]. The alignment served as input for RAxML [83] for phylogenetic tree inference using PROTGAMMAAUTO as model and performing rapid bootstrap analysis with 100 cycles as well as best-scoring ML tree search. This yielded a GAMMA+LG model with empirical base frequencies. The resulting tree was further visualized with iTOL [84].

Bacteroides species containing TnaA were extracted from the total amount of Bacteroides species in the analyzed 16S samples (**Table B in S1 Table**) since it was reported that not all Bacteroides species contain TnaA enzyme [85]. The analysis was done with the statistics software R (R version 3.6.1, https://www.R-project.org/). First, list of reviewed TnaAs was downloaded from Uniprot (https://www.uniprot.org/), and a BLAST [79] search was locally carried out against the translated HMP Reference Genome (HMRGD) sequence database, which was subsetted to contain only GI tract as body site. Blastp was run in default configuration, and hits with less than 90% query cover or less than 30% identity were discarded to still stay in the safe space for homology modelling. The resulting sequencing was compared to Bacteroides species detected in 16S rRNA analysis (**Table B in S1 Table**). This resulted in the list of Bacteroides (tnaA-encoding), the rest was annotated as Bacteroides (non-tnaA-encoding).

### Rats and total gut transit time measurements

All animal procedures were approved by the Groningen University Committee of Animal experiments (approval number: AVD1050020197786) and were performed in adherence to the NIH Guide for the Care and Use of Laboratory Animals.

Sixteen male adult WTG rats (Groningen breed, male, age 19 to 23 weeks) housed 3 to 5 animals/cage had ad libitum access to water and food (Altromin 1414 mod.—NL_141005) in a temperature (21 ± 1 °C) and humidity-controlled room (45% to 60% relative humidity), with a 12-h light/dark cycle (lights off at 1:00 PM). These outbred rats are very frequently used in behavioral studies due to the high interindividual variation [86], thus resembling, to some extent, the human interindividual variation. On 3 occasions over a period of 1 week, rats were taken from their social housing cage in the beginning of the dark-phase cycle and put in an individual training cage (L × W × H = 25 × 25 × 40 cm) containing wire-mesh, without bedding, food, or water. Ten minutes after transfer to these cages, rats were given a drinking pipette with a 2.5-mL sucrose solution (10% w/v). On 2 additional training occasions, rats were placed in metabolic cages, where carmine red test was conducted, and were trained as described above. Over the course of training, all rats were trained to drink the sucrose solution avidly. On the eighth occasion, the 10% sucrose solution was used as a vehicle for the 1.2% carmine red (C1022, Sigma) and 0.5% methylcellulose (M0512, Sigma) mixture. All rats were given 1.2 mL of the carmine red solution to measure baseline TGTT before they were randomly appointed to either vehicle (10% sucrose) group or 5-HI-treated group. After baseline measurements of TGTT, rats from 5-HI-treated group were given daily 30 mg/kg of 5-HI (H31859, Sigma) in drinking pipette with a 2.5-mL sucrose solution (10% w/v) for a period of 11 days. Rats from the vehicle group were treated for the same period of 11 days, but 5-HI was not added to the sucrose solution. A total of 10% sucrose solvent was used only for in vivo experiment as described before [1]. A total of 30 mg/kg of 5-HI was chosen based on previous report [87]. Fecal pellets were monitored every 30 min for the presence of carmine red in the fecal pellet. Total time taken for the appearance of the first red pellet was recorded as the TGTT.

### 5-HT release experiment

RIN14B (ATCC; Cat# CRL-2059) cells were seeded in 12-well plates at the rate of 2 × 10^5^ cells/1 mL in RPMI1640 containing 10% FBS/well and cultured for 72 h. The medium was removed before washing the cells with HBSS (+Ca^2+^, +Mg^2+^) containing 0.1% BSA and 2 μM fluoxetine (F132, Sigma) (HBSS-S). The HBSS-S was again removed and replaced with 0.25 mL HBSS-S containing 100 μM 5-HI or only 0.25 mL HBSS-S, after which the solution was incubated further for 30 min at 37 °C. The supernatants were collected and stored at −20 °C until 5-HT measurement using an enzyme immunoassay (EIA) kit (Abnova).

### Intestinal assay for conversion of 5-HI

Rat intestinal tissues were collected from WTG male rats (*n* = 3) and rinsed with 1X phosphate saline buffer. Tissue extracts were prepared by homogenization in Mini-Beadbeater and centrifuged at 13,000 × g (4 °C) for 20 min. Total protein concentration was determined by Bradford assay. Intestinal assay was performed at 37 °C and with 50 μM 5-HI. Incubations were conducted at 1 mg/mL of intestinal tissue extracts in 50 mM phosphate buffer (pH 7.4). Reactions were initiated by addition of 5-HI to prewarmed reaction mixture. A total of 100 μL samples were collected and quenched in 400 μL ice-cold 100% methanol at 0 and 90 min and 24 h after initiation. Samples were stored at −20 °C until analysis by HPLC-UV.

### Organ-bath experiments

WTG male rats were killed, and a proximal colon was immediately removed and washed in 1X PBS and placed in 0.7% NaCl solution. Approximately 3 mm rings were cut and were placed in an organ bath (Tissue Bath Station with SSL63L force transducer, Biopac Systems Inc., Varna, Bulgaria) filled with Krebs–Henseleit solution (NaCl, 7.02 g/L; KCl, 0.44 g/L; CaCl_2_.2H_2_O, 0.37 g/L; MgCl_2_.6H_2_O, 0.25 g/L; NaH_2_PO_4_.H_2_O 0.17 g/L; Glucose, 2.06 g/L; NaHCO_3_, 2.12 g/L) gassed with Carbogen gas mixture (5% CO_2_, balanced with O_2_) at 37 °C. At the beginning of the experiment, tension of the intestine of 0.5 to 1 g was obtained by adjusting the stretcher. Under these conditions, colonic rings were equilibrated for at least 45 to 60 min with replacement of Krebs–Henseleit solution approximately every 15 min. Sequentially, 10 μM serotonin (5-HT) (H9523, Sigma) or 50 μM ACh (A2661, Sigma) was added to induce a specific contractile response, and it was followed by addition of 100 μM 5-HI (H31859, Sigma). All the studied compounds added to the intestinal tissue in the organ bath were dissolved in MilliQ-filtered water unless otherwise noted. To perform the tests with antagonists for the receptors involved in the gut motility, either 1 μM ondansetron (5-HT_3_ antagonist; O3639, Sigma), 1 μM SB-207266 (5-HT_4_ antagonist; SML1349, Sigma), 1 μM TTX (sodium ion transport blocker on the enteric neurons; TTX; 1078/1, Tocris Bioscience), 1 μM hexamethonium bromide (a nicotinic cholinergic antagonist; 4111, Tocris Bioscience), 1 μM AQ-RA 741 (muscarinic receptor 2 antagonist (M2R); 2292, Tocris Bioscience), 100 nM DAU 5884 hydrochloride (M3R antagonist; 2096; Tocris Bioscience), 1 μM ML204 (TRPC4 antagonist; 4732, Tocris Bioscience), 1 μM SAR 7334 (TRPC6 antagonist; 5831; Tocris Bioscience), 100 μM ATP (A1335; Duchefa Bioscience), or 1 μM nifedipine (L-type Ca^2+^ channels antagonist; 1075; Tocris Bioscience) was applied sequentially with either ACh, 5-HT, or 5-HI. As control, 0.1% DMSO (solvent of AQ-RA 741, ML 204, and nifedipine) or 20 mM citrate buffer (solvent of TTX) was applied prior to addition of mentioned compounds to the tissue to check for any change in contractions. Each treatment lasted for approximately 10 min. Data were recorded using BioPac Student Lab 4.1 (Build: February 12, 2015). Quantitative analysis of the organ bath recordings was performed in BioPac Student Lab 4.1, where each 10-min recording segment was selected and FFT analysis was done with following settings: Data were padded with zeros, mean was removed, magnitude was displayed with linear transform, and signal was processed using Hamming window. Afterwards, the maximum amplitudes of the dominant frequencies obtained from FFT analysis were selected and analyzed in GraphPad Prism 7. The illustrative contractions (5 min) segments data were extracted from BioPac Student Lab 4.1 and analyzed in GraphPad Prism 7.

### Statistical analysis and (non)linear regression models

All statistical tests and (non)linear regression models were performed using GraphPad Prism 7. For enzyme kinetics with 5-HTP as substrate, the nonlinear Michaelis–Menten regression model was used. For enzyme kinetics with tryptophan as substrate, the nonlinear substrate inhibition kinetic model was used. For pairwise and 2 independent group comparison unpaired *t* test was used, while for multiple group comparison, 1-way-ANOVA or RM 2-way ANOVA was performed followed by a Fisher LSD test. The microbiota composition was statistically compared using Wilcoxon rank sum test for all the taxa and each group **(Table J in S1 Table**). For ex vivo organ-bath measurements, the Wilcoxon matched-pairs (before/after) signed rank test was used. Data are presented as mean ± SEM, and *p* < 0.05 was considered statistically significant. The (n) refers to the number of individuals, rats, or rats’ tissues used for each experiment. The number of rats for in vivo experiment was based on previous study [88] to achieve power of 80% with α of 0.05. Specific test, significance, and (n) number are indicated in the Fig legends.

## Supporting information

S1 Fig Gut bacteria convert 5-HTP to 5-HI. **(A)** Formation of 5-HI was identified and confirmed by LC-MS. Chromatograms show mass [M+H]^+^ m/z = 134,0602, which corresponds to exact molecular weight of 5-HI in positive mode. (**B)** No basal levels of 5-HTP were detected in the control samples (gray line) in the fecal samples from healthy individuals at time point 0 h. (**C**) Chromatogram shows that tryptophan was not converted to indole in the fecal samples of the Non-Converters group. The raw data of **B** and **C** can be found in S1 Raw images. 5-HI, 5-hydroxyindole; 5-HTP, 5-hydroxytryptophan; LC-MS, Liquid Chromatography–Mass Spectrometry.
(TIF)

S2 Fig Bacterial TnaA is responsible for the conversion of 5-HTP. **(A)** Overnight culture of *E*. *coli* BW25113^WT^ (black line) and *E*. *coli* BW25113^*ΔtnaA*^ (red line) incubated at 37 °C with agitation with Trp present in the culture medium for 24 h. Curves represent 1 example of 3 biological replicates. The raw data can be found in S2 Raw images. (**B)** High levels of Trp do not prevent the production of 5-HI in the fecal samples after 24 h of anaerobic incubation. (**C)** Cell lysates of *B*. *thetaiotamicron* VPI-5482 incubated at 37 °C converted 5-HTP to 5-HI after 48 h. The raw data of **B** and **C** can be found in S3 Raw images. 5-HI, 5-hydroxyindole; 5-HTP, 5-hydroxytryptophan; Trp, tryptophan.
(TIF)

S3 Fig MSA of TnaA from several human gut bacterial species. An MSA of the 15 closest orthologs of *E*. *coli*, *F*. *nucleatum*, and *B*. *thetaiotamicron*. Levels of the conserved amino acids are highlighted in the shades of blue (dark blue = higher level of conservation, light blue = lower level of conservation). The level of conservation in the multiple active sites of TnaA is highlighted in red. MSA, multiple sequence alignment; TnaA, tryptophanase.
(TIF)

S4 Fig Bacterial production of 5-HI is dependent on the microbiota composition and pH levels. **(A)** Pie charts represent the difference in relative percentage of Bifidobacterium genus annotated in light blue (not exploded). Exploded slices represent the difference in tnaA-encoding bacterial phyla among High, Intermediate, and Non-Converters. Slices annotated in pink (not exploded) represent sum of the Bacteroides species which do not contain TnaA enzyme. Parts annotated in the shades of gray represent the rest of bacterial genera detected in the samples. (**B)** PCA score plot indicates clear separation of High Converters from Intermediate and Non-Converters group. Two PCs explained 43.1% and 14.9% of total variances in 16S rRNA sequencing data. Only the first 5 top most contributing species are shown. (**C)** RDA plot indicates significant influence of 5-HI levels, being the explanatory variable (*p*-value = 0.017). Only the first 5 top most contributing species are shown. (**D)** Graph shows OD_600_ measurements of *F*. *nucleatum* subsp. *animalis* cultured anaerobically at 37 °C in Enriched Beef Broth adjusted to different pH. (**E)** Graph shows the relative 5-HI concentration produced in the *F*. *nucleatum* subsp. *animalis* lysates after 48 h of incubation with 100 μM 5-HTP at different pH levels. (**F)** Graph shows the relative 5-HI concentration produced in the *B*. *thetaiotamicron* lysates after 48 h of incubation with 100 μM 5-HTP at different pH levels. The raw data used for quantification of **D, E,** and **F** can be found in S1 Fig Data. 5-HI, 5-hydroxyindole; 5-HTP, 5-hydroxytryptophan; PCA, principal component analysis; RDA, redundancy analysis; TnaA, tryptophanase.
(TIF)

S5 Fig 5-HI and its effect on colonic contractility. **(A)** Bar graph represents remaining percentage of 5-HI after incubation for 90 min and 24 h in rat colonic tissue extracts (*n* = 3). Error bars demonstrate SEM. **(B–E)** Bar graphs and illustrative recordings representing the inhibitory effect of either (**B)** 1 μM ondansetron, (**C)** 1 μM SB-207266, (**D)** 1 μM TTX, or (**E)** 1 μM hexamethonium on the 5-HT-induced response. (**F**) Bar graph represents no inhibitory effect of 1 μM hexamethonium on 5-HI-induced response. **(G–I)** Bar graphs represent an inhibitory effect on ACh-induced response by the addition of either **(G)** 1 μM AQ-RA 741, (**H)** 100 nM DAU 5884 hydrochloride, or (**I)** 1 μM ML 204 and SAR 7334. (**J)** Bar graph and its representative recording of colonic contractions showing no inhibitory effect on 5-HI-induced response when 1 μM ML 204 together with 1 μM SAR 7334 were added. (**K)** Bar graph represents contractile agent (ATP) acting independently of L-type Ca^2+^ channels. Data represent 3–5 biological replicates. Data were analyzed using the Wilcoxon matched-pairs (before/after) signed rank test (*p < 0.05; ***p < 0.001). Error bars represent SEM. Quantitative analysis of the organ bath data is described in Materials and method section. The raw data used for quantification of **A–K** can be found in S2 Fig Data.
(TIF)

S1 Data The raw data used for quantification of Fig 1A (lower panel) and Fig 1B. (XLSX)

S2 Data The raw data used for quantification of Fig 2C and 2D. (XLSX)

S3 Data The raw data used for quantification of Fig 4A, 4B and 4C. (XLSX)

S4 Data The raw data used for quantification of Fig 5A, 5B, 5C, 5D, 5E, 5F, 5G, 5H, 5I, 5J and 5K. (XLSX)

S1 Raw images The raw data of Fig 1A. (PDF)

S2 Raw images The raw data of Figs 2B, 2E, 2F and S2A. (PDF)

S3 Raw images The raw data of Figs 3B, 3C, 3D and S2B and S2C. (PDF)

S1 Fig Data The raw data used for quantification of S4D, S4E and S4F Fig. (TIF)

S2 Fig Data The raw data used for quantification of S5A, S5B, S5C, S5D, S5E, S5F, S5G, S5H, S5I, S5J and S5K Fig. (TIF)

S1 Table Contains Table A (Tryptophanase encoding orthologues of E. coli BW25113, Fusobacterium nucleatum ATCC 51191, and B. thetaiotamicron VPI-5482), Table B (16S rRNA microbial profiling), Table C (Supporting data for PCA and RDA analyses), Table D (Pearson R correlations between 5-HI levels and tnaA-encoding bacteria), Table E (Constituents of Liquid Amies + 20% Glycerol medium), Table F (Constituents of Enriched Beef Broth medium), Table G (Bacterial strains), Table H (Primers), Table I (Plasmids), and Table J (Microbiota statistical analysis using Wilcoxon rank sum test for all the taxa and each group (unadjusted and adjusted p-values).
(XLSX)

## Abbreviations:

5-HI: 5-hydroxyindole
5-HT: serotonin
5-HTP: 5-hydroxytryptophan
ACh: acetylcholine
ENS: enteric nervous system
GI: gastrointestinal
HPLC-ED/UV: High-Performance Liquid Chromatography coupled with Electrochemical detection and UV detection
LC-MS: Liquid Chromatography–Mass Spectrometry
L-VDCCs: L-type voltage-dependent Ca2+ channels
M2R: muscarinic acetylcholine receptor subtype 2
M3R: muscarinic acetylcholine receptor subtype 3
PCA: principal component analysis
RDA: redundancy analysis
TGTT: total gut transit time
TnaA: tryptophanase
TRPC4: transient receptor potential channel 4
TRPC6: transient receptor potential channel 6
TTX: tetrodotoxin
WTG: wild-type Groningen
