# Supplementary figures and images for: Gut bacteria-derived 5-hydroxyindole is a potent stimulant of intestinal motility via its action on L-type calcium channels
Source: PLoS Biol. 2021 Jan 22;19(1):e3001070. doi: 10.1371/journal.pbio.3001070 (PMC7857600; doi:10.1371/journal.pbio.3001070)

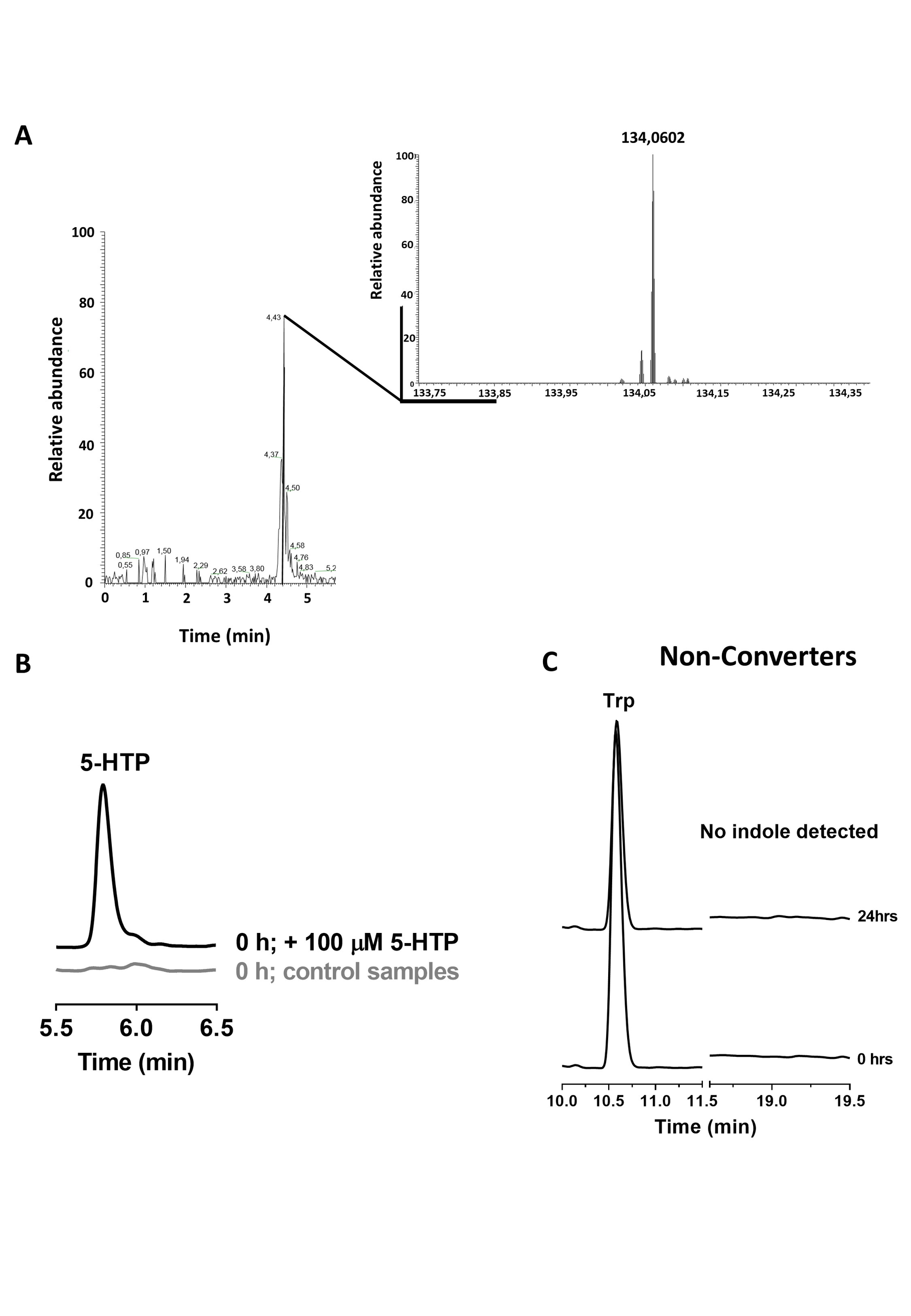

Supplement: S1 Fig — (A) Formation of 5-HI was identified and confirmed by LC-MS. Chromatograms show mass [M+H]+ m/z = 134,0602, which corresponds to exact molecular weight of 5-HI in positive mode. (B) No basal levels of 5-HTP were detected in the control samples (gray line) in the fecal samples from healthy individuals at time point 0 h. (C) Chromatogram shows that tryptophan was not converted to indole in the fecal samples of the Non-Converters group. The raw data of B and C can be found in S1 Raw images. 5-HI, 5-hydroxyindole; 5-HTP, 5-hydroxytryptophan; LC-MS, Liquid Chromatography–Mass Spectrometry. (TIF) [file pbio.3001070.s001.tif]

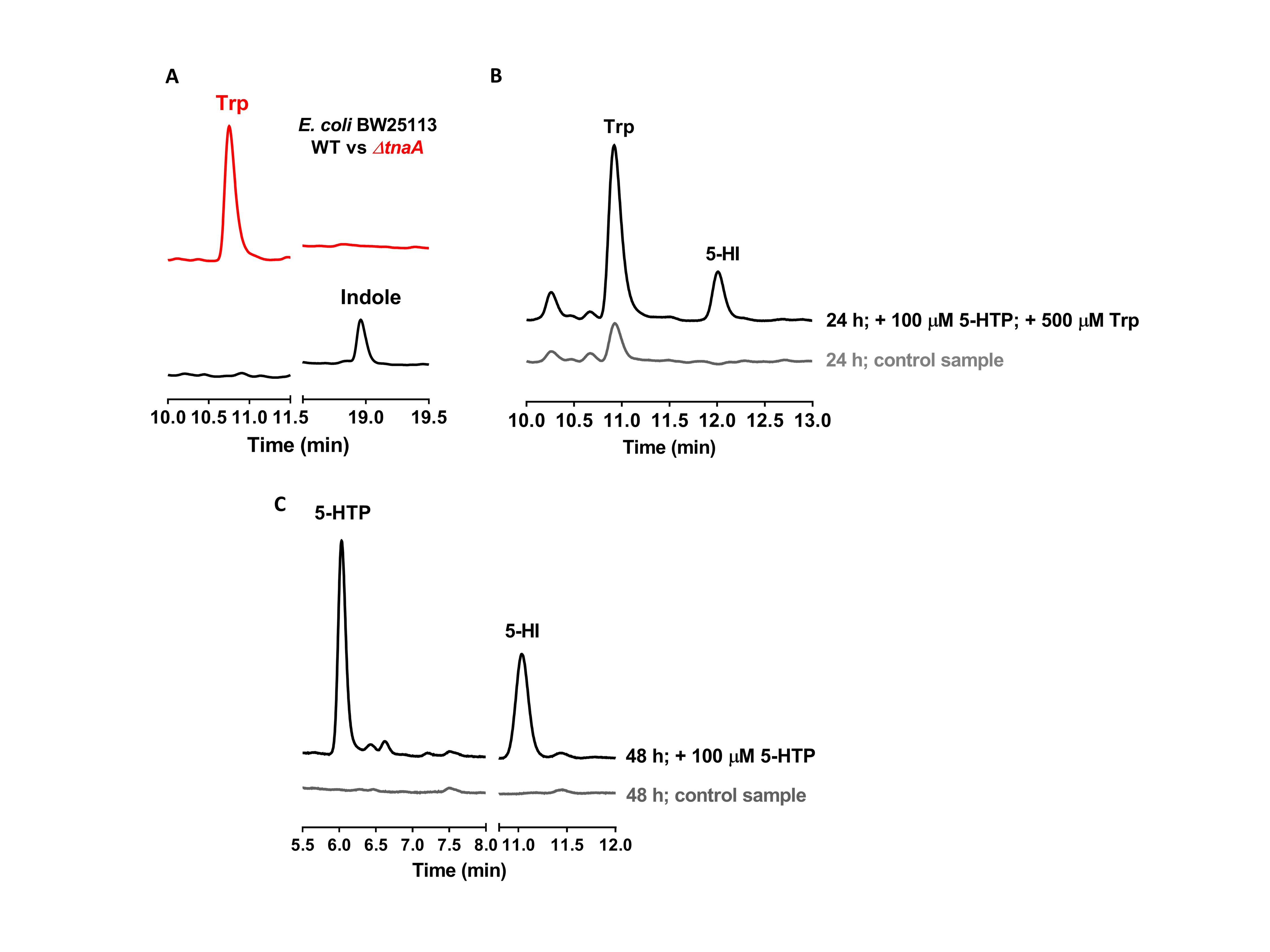

Supplement: S2 Fig — (A) Overnight culture of E. coli BW25113WT (black line) and E. coli BW25113ΔtnaA (red line) incubated at 37 °C with agitation with Trp present in the culture medium for 24 h. Curves represent 1 example of 3 biological replicates. The raw data can be found in S2 Raw images. (B) High levels of Trp do not prevent the production of 5-HI in the fecal samples after 24 h of anaerobic incubation. (C) Cell lysates of B. thetaiotamicron VPI-5482 incubated at 37 °C converted 5-HTP to 5-HI after 48 h. The raw data of B and C can be found in S3 Raw images. 5-HI, 5-hydroxyindole; 5-HTP, 5-hydroxytryptophan; Trp, tryptophan. (TIF) [file pbio.3001070.s002.tif]

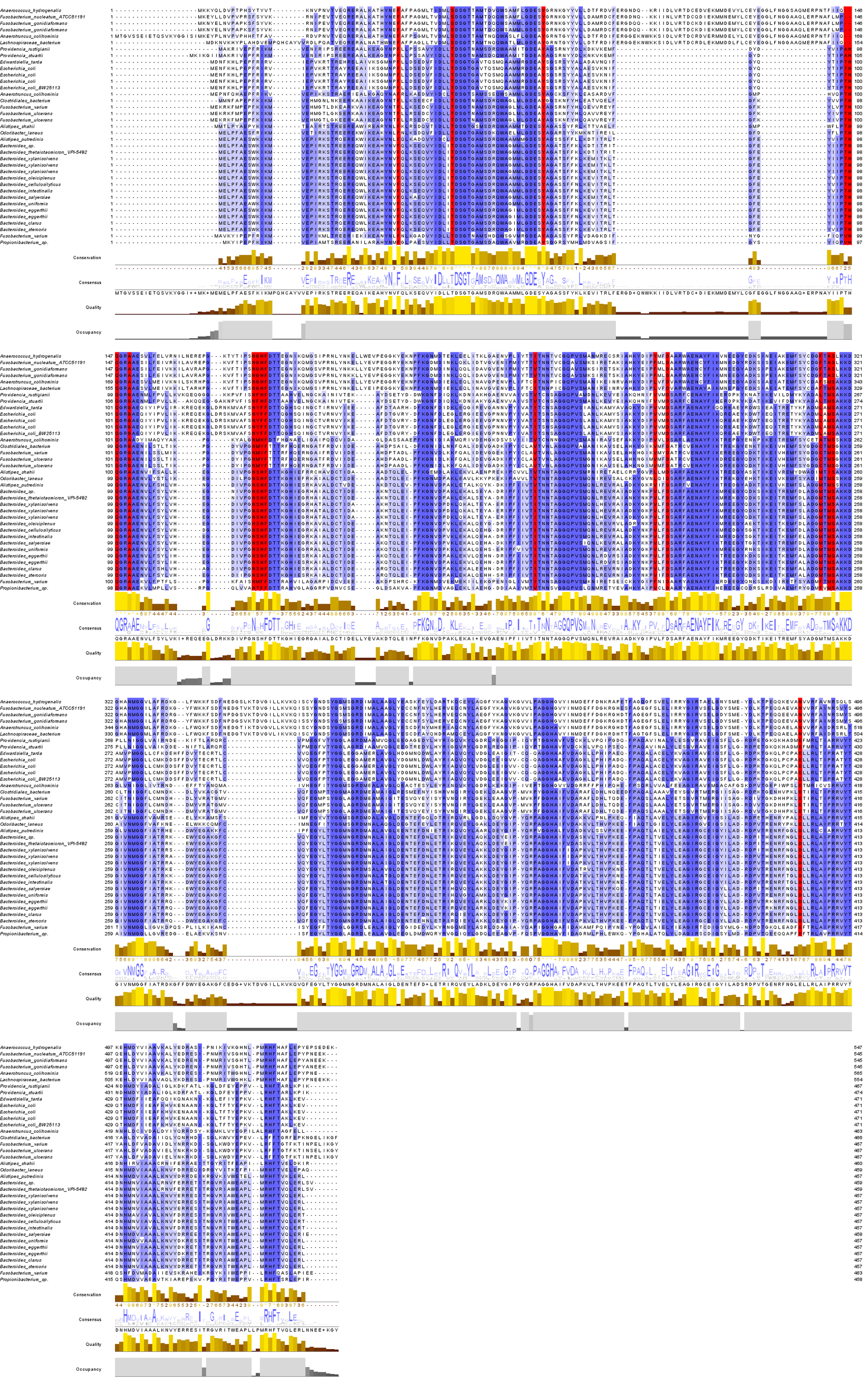

Supplement: S3 Fig — An MSA of the 15 closest orthologs of E. coli, F. nucleatum, and B. thetaiotamicron. Levels of the conserved amino acids are highlighted in the shades of blue (dark blue = higher level of conservation, light blue = lower level of conservation). The level of conservation in the multiple active sites of TnaA is highlighted in red. MSA, multiple sequence alignment; TnaA, tryptophanase. (TIF) [file pbio.3001070.s003.tif]

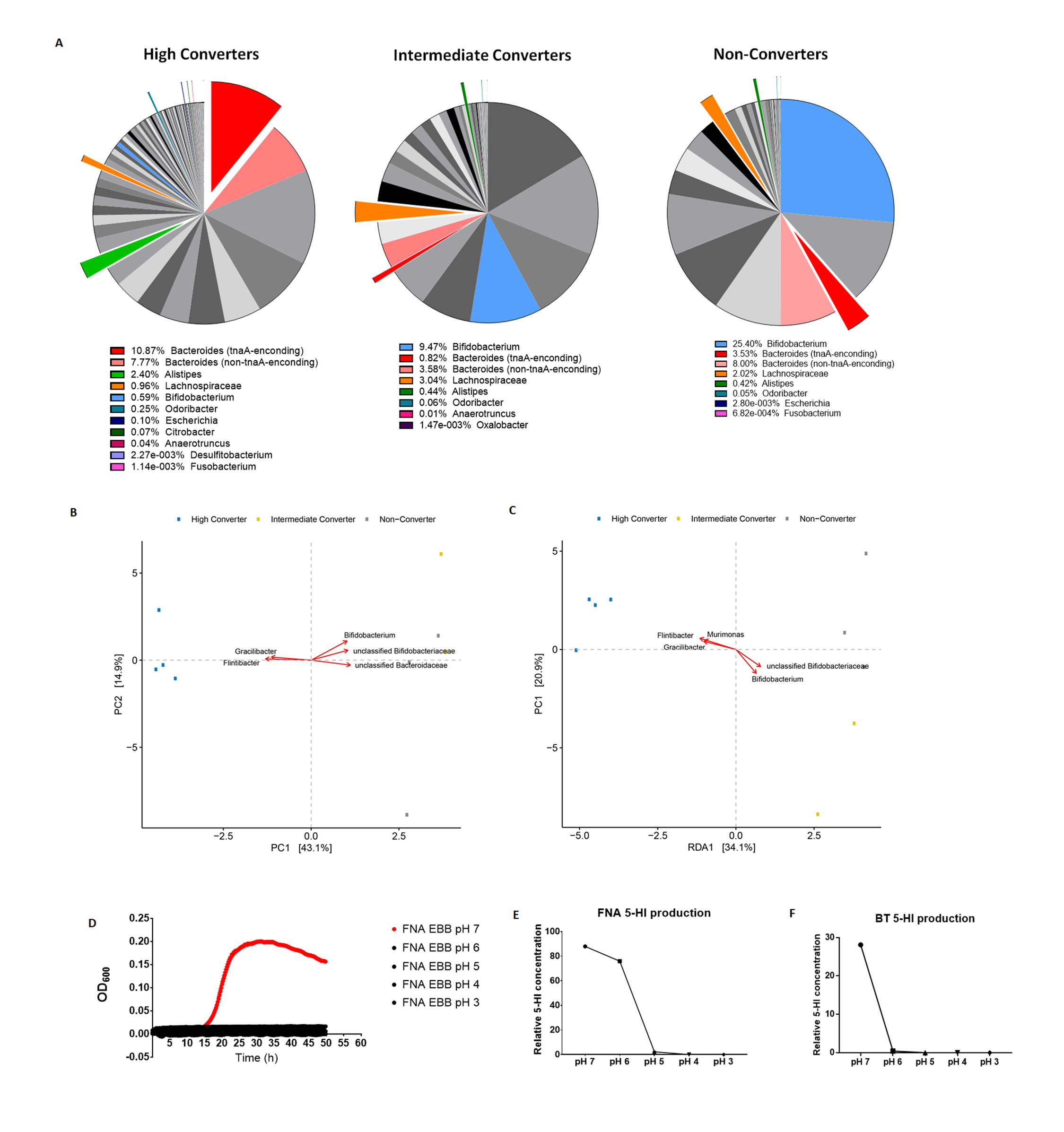

Supplement: S4 Fig — (A) Pie charts represent the difference in relative percentage of Bifidobacterium genus annotated in light blue (not exploded). Exploded slices represent the difference in tnaA-encoding bacterial phyla among High, Intermediate, and Non-Converters. Slices annotated in pink (not exploded) represent sum of the Bacteroides species which do not contain TnaA enzyme. Parts annotated in the shades of gray represent the rest of bacterial genera detected in the samples. (B) PCA score plot indicates clear separation of High Converters from Intermediate and Non-Converters group. Two PCs explained 43.1% and 14.9% of total variances in 16S rRNA sequencing data. Only the first 5 top most contributing species are shown. (C) RDA plot indicates significant influence of 5-HI levels, being the explanatory variable (p-value = 0.017). Only the first 5 top most contributing species are shown. (D) Graph shows OD600 measurements of F. nucleatum subsp. animalis cultured anaerobically at 37 °C in Enriched Beef Broth adjusted to different pH. (E) Graph shows the relative 5-HI concentration produced in the F. nucleatum subsp. animalis lysates after 48 h of incubation with 100 μM 5-HTP at different pH levels. (F) Graph shows the relative 5-HI concentration produced in the B. thetaiotamicron lysates after 48 h of incubation with 100 μM 5-HTP at different pH levels. The raw data used for quantification of D, E, and F can be found in S1 Fig Data. 5-HI, 5-hydroxyindole; 5-HTP, 5-hydroxytryptophan; PCA, principal component analysis; RDA, redundancy analysis; TnaA, tryptophanase. (TIF) [file pbio.3001070.s004.tif]

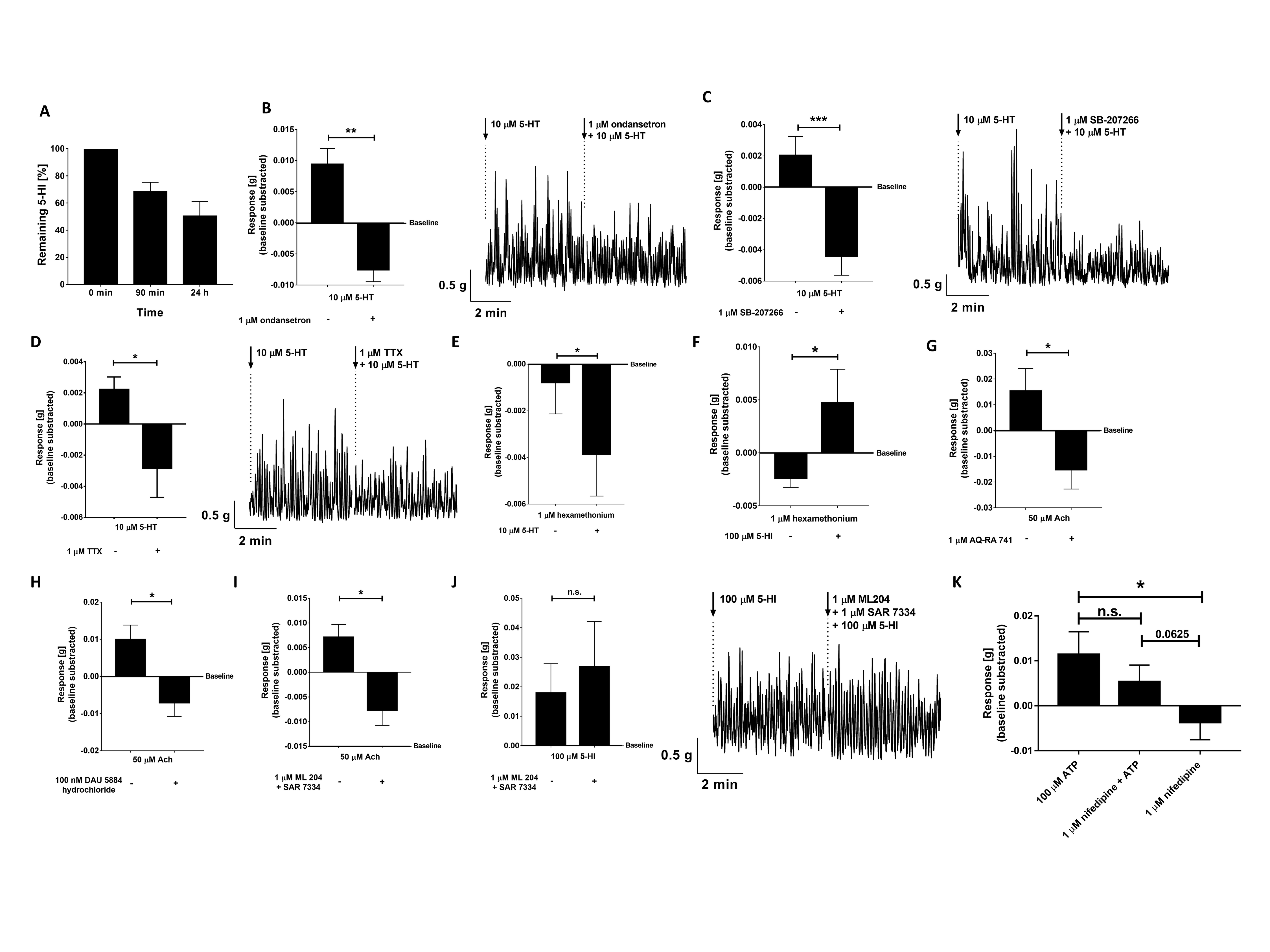

Supplement: S5 Fig — (A) Bar graph represents remaining percentage of 5-HI after incubation for 90 min and 24 h in rat colonic tissue extracts (n = 3). Error bars demonstrate SEM. (B–E) Bar graphs and illustrative recordings representing the inhibitory effect of either (B) 1 μM ondansetron, (C) 1 μM SB-207266, (D) 1 μM TTX, or (E) 1 μM hexamethonium on the 5-HT-induced response. (F) Bar graph represents no inhibitory effect of 1 μM hexamethonium on 5-HI-induced response. (G–I) Bar graphs represent an inhibitory effect on ACh-induced response by the addition of either (G) 1 μM AQ-RA 741, (H) 100 nM DAU 5884 hydrochloride, or (I) 1 μM ML 204 and SAR 7334. (J) Bar graph and its representative recording of colonic contractions showing no inhibitory effect on 5-HI-induced response when 1 μM ML 204 together with 1 μM SAR 7334 were added. (K) Bar graph represents contractile agent (ATP) acting independently of L-type Ca2+ channels. Data represent 3–5 biological replicates. Data were analyzed using the Wilcoxon matched-pairs (before/after) signed rank test (*p < 0.05; ***p < 0.001). Error bars represent SEM. Quantitative analysis of the organ bath data is described in Materials and method section. The raw data used for quantification of A–K can be found in S2 Fig Data. (TIF) [file pbio.3001070.s005.tif]

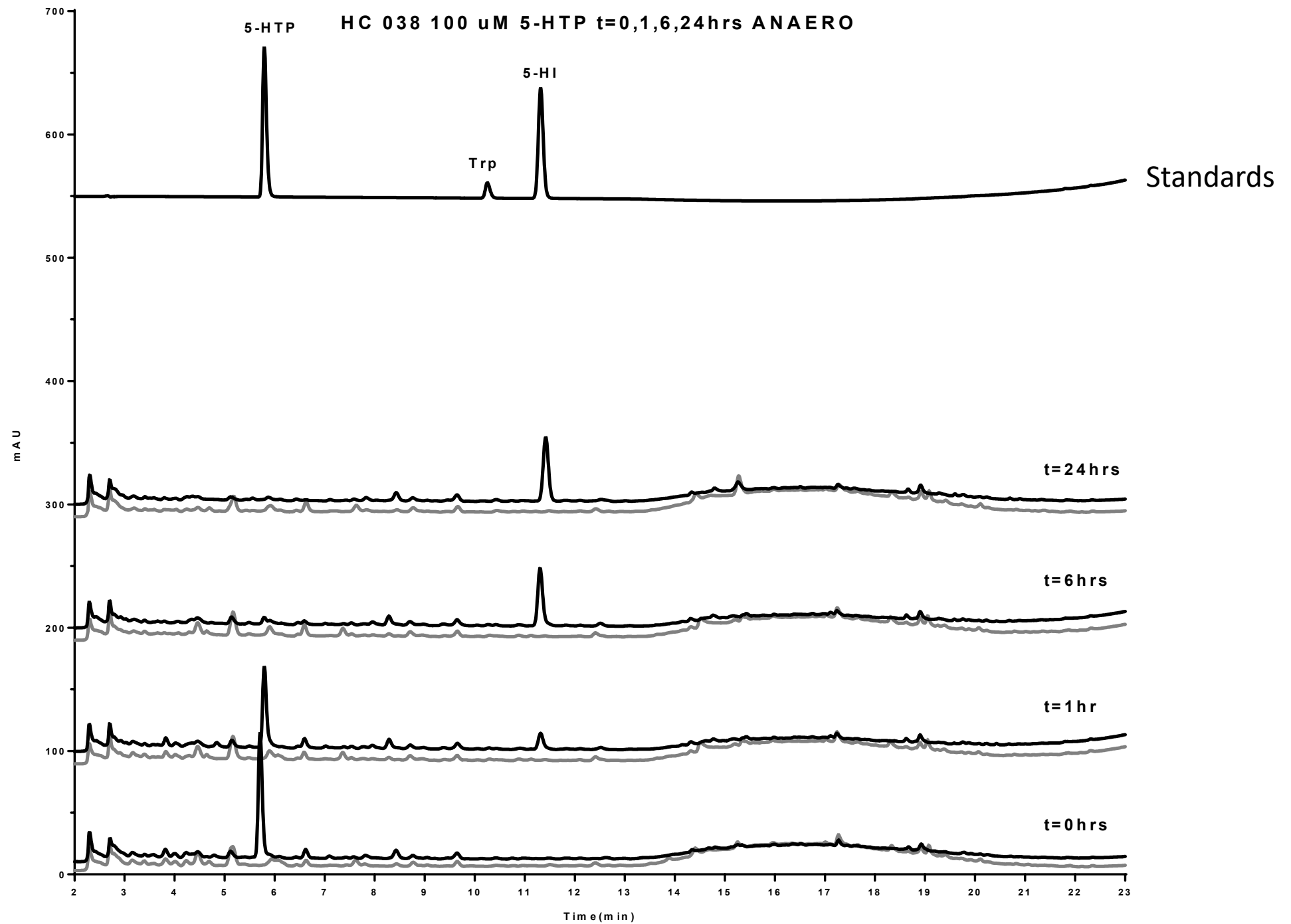

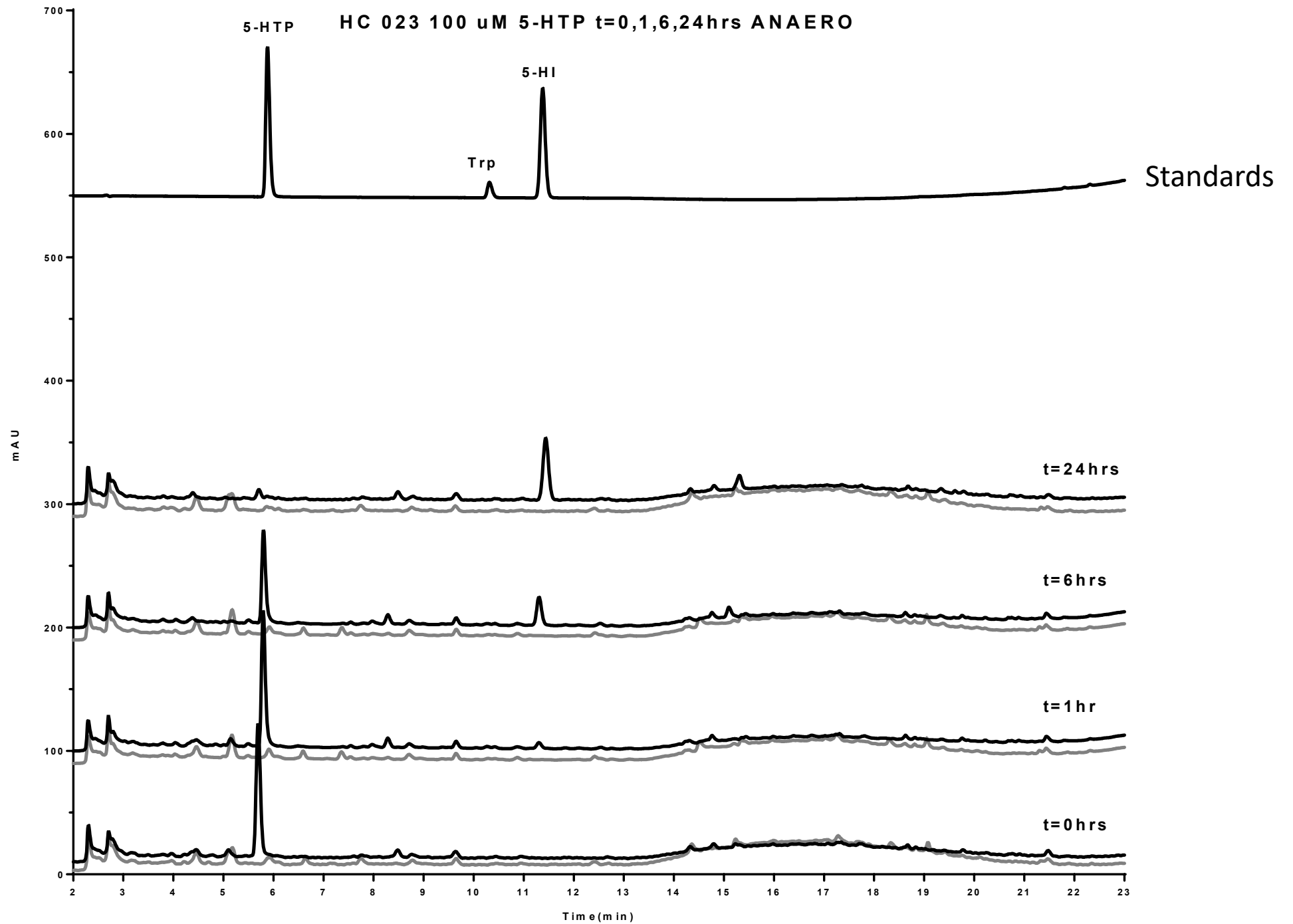

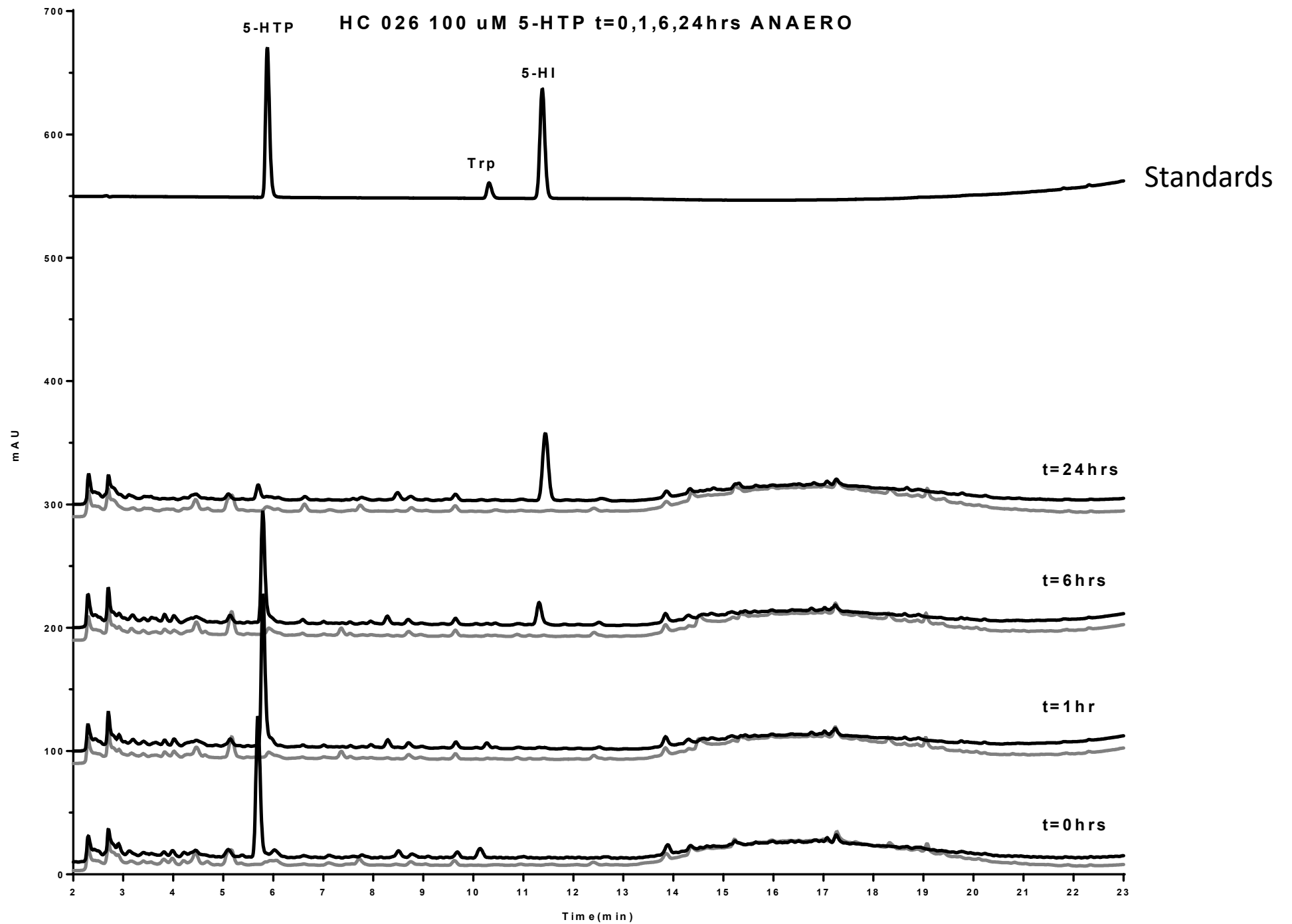

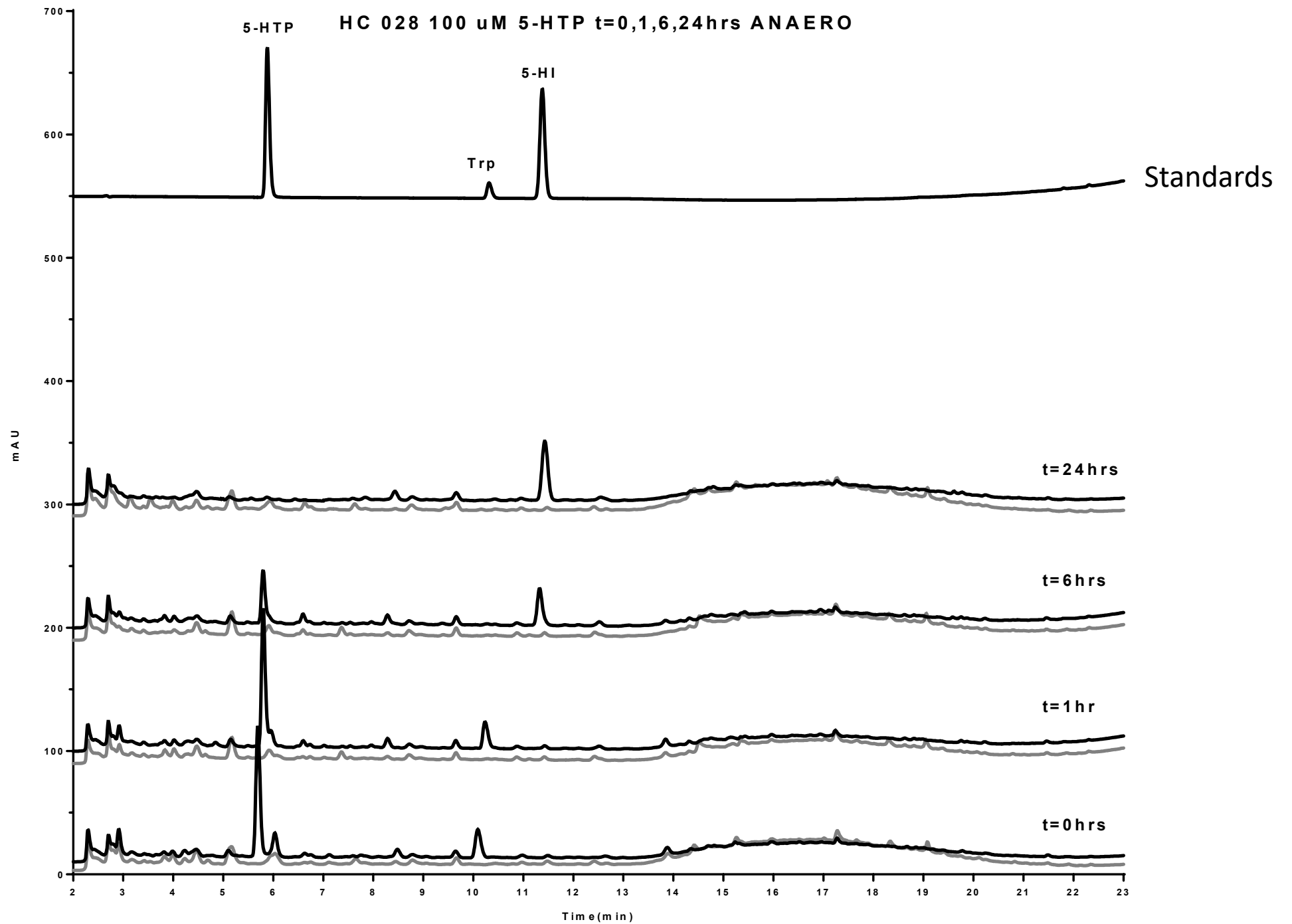

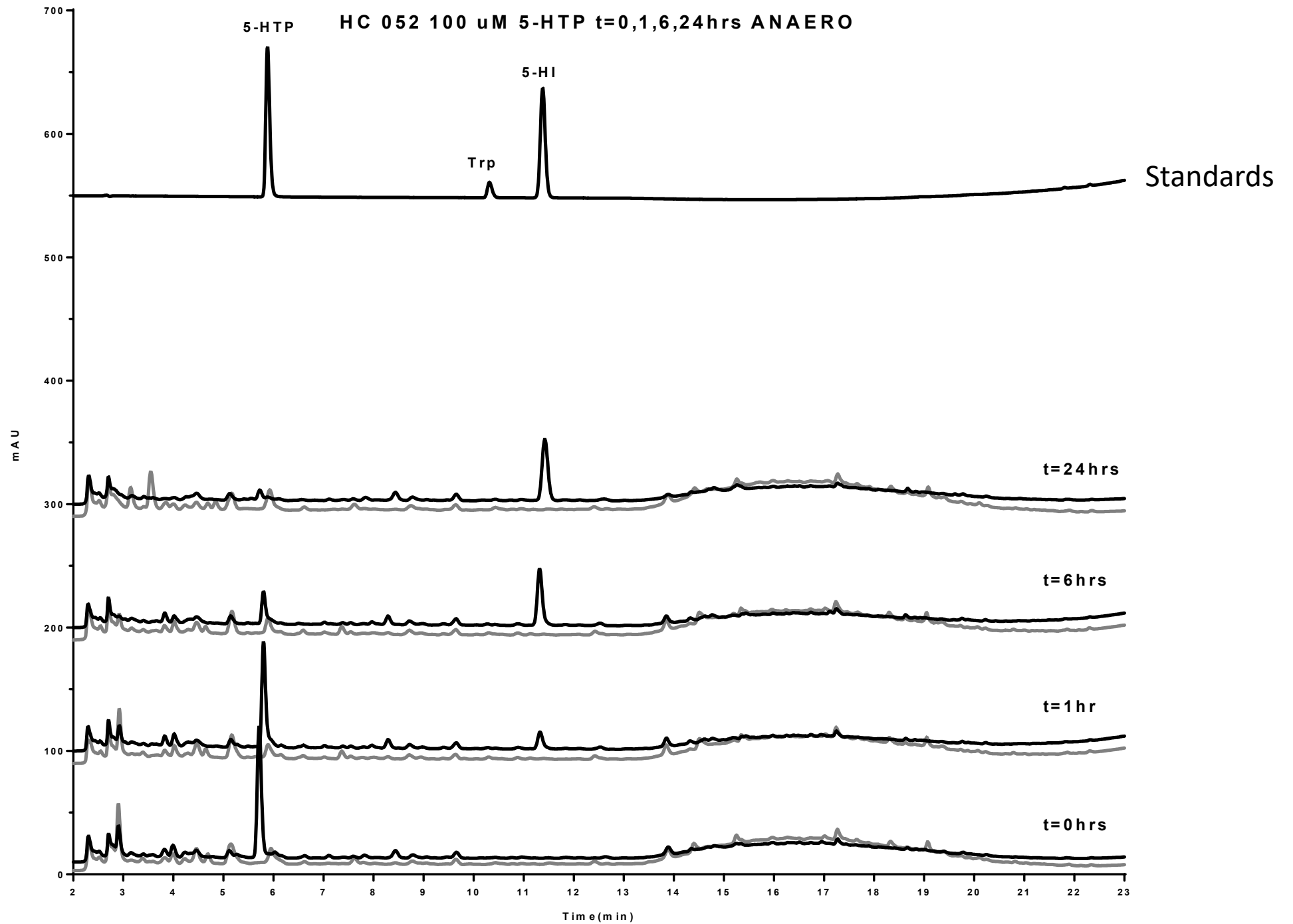

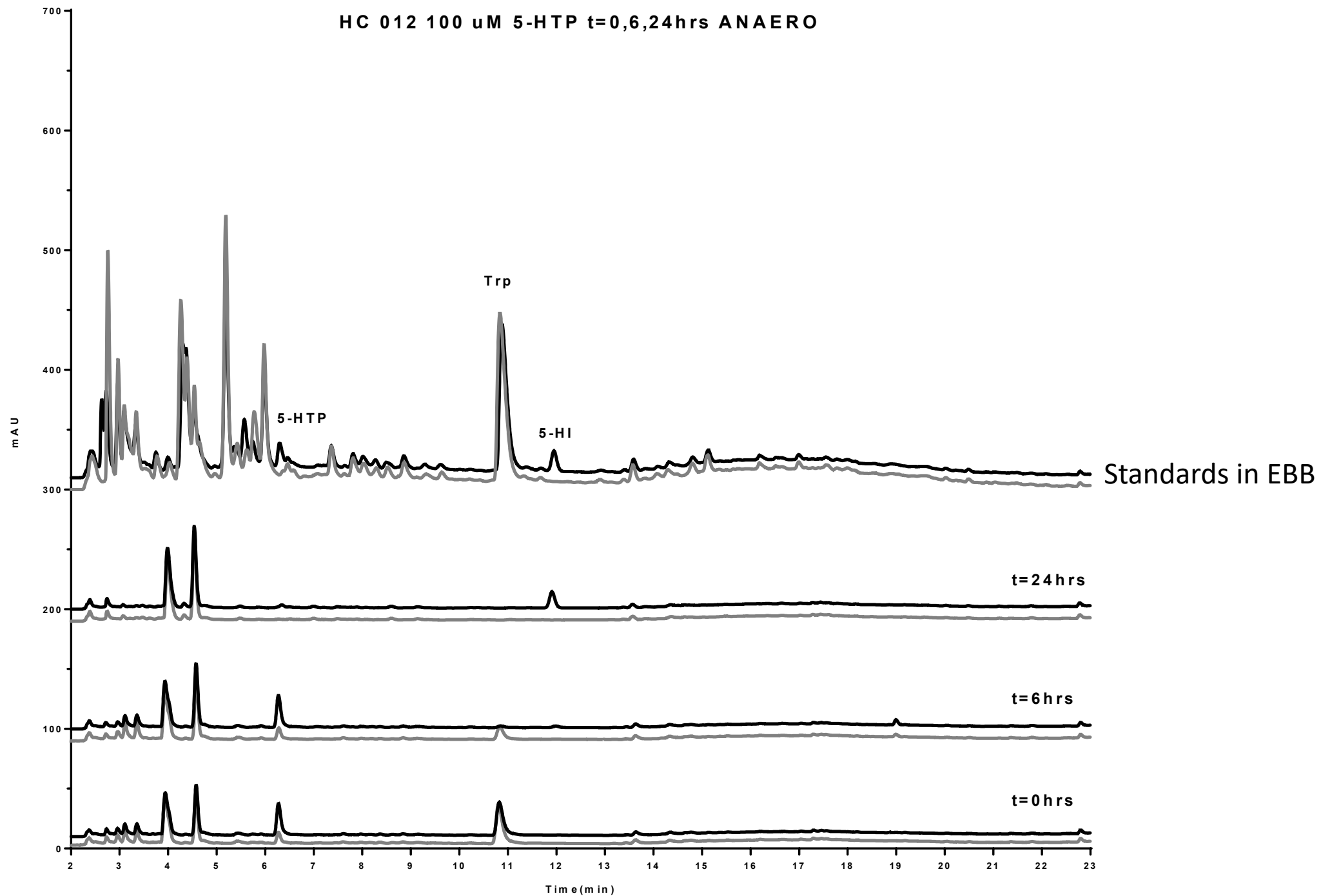

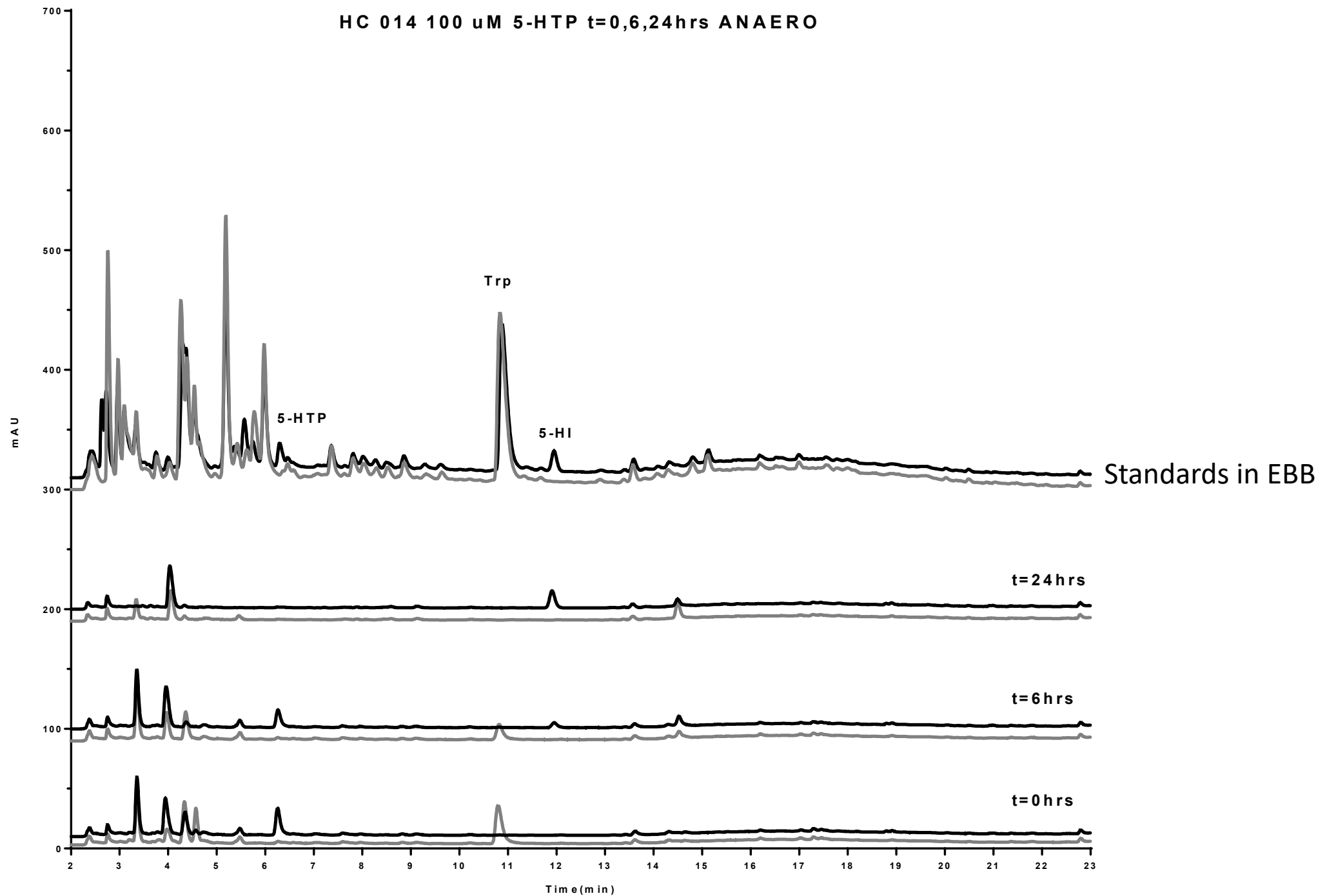

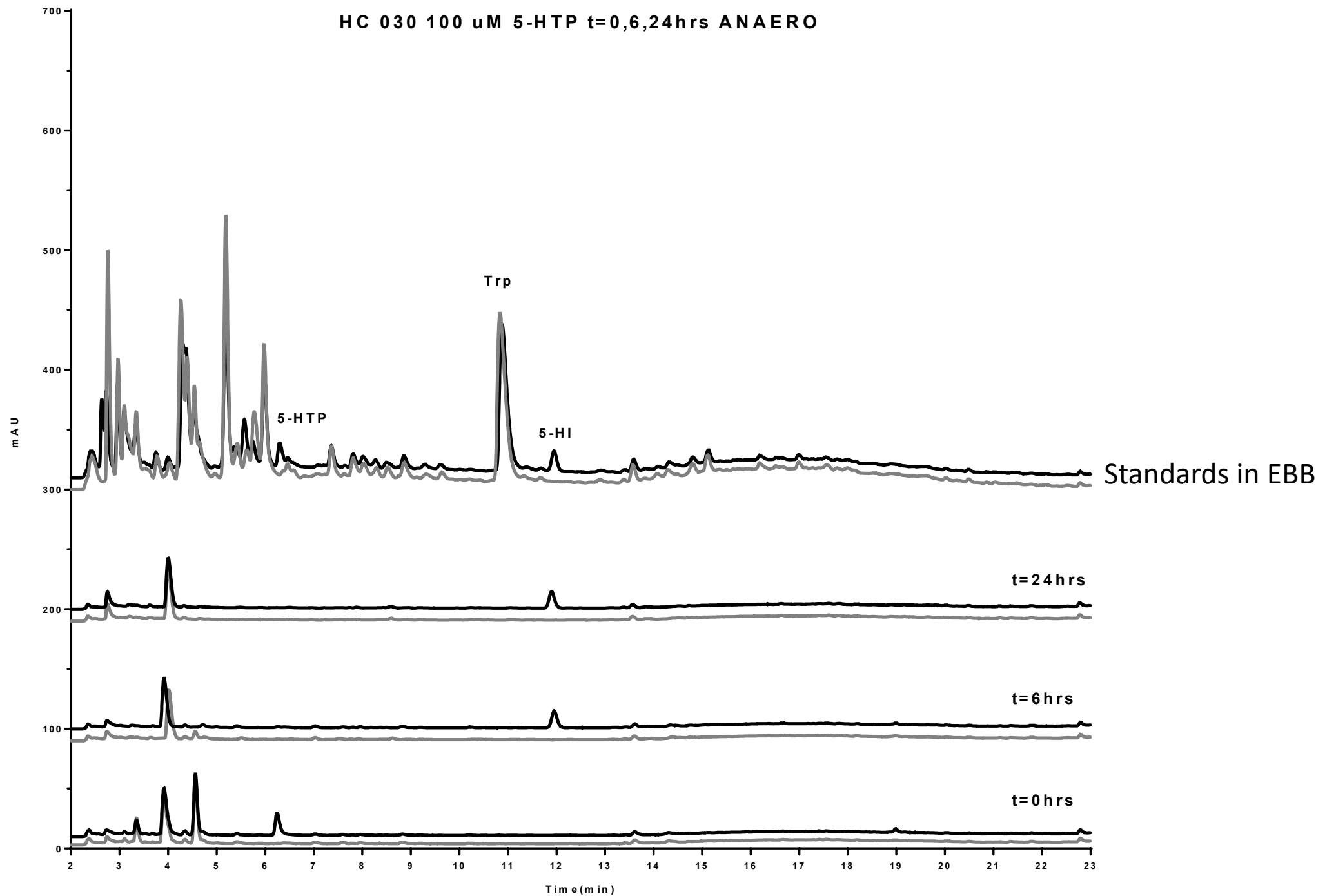

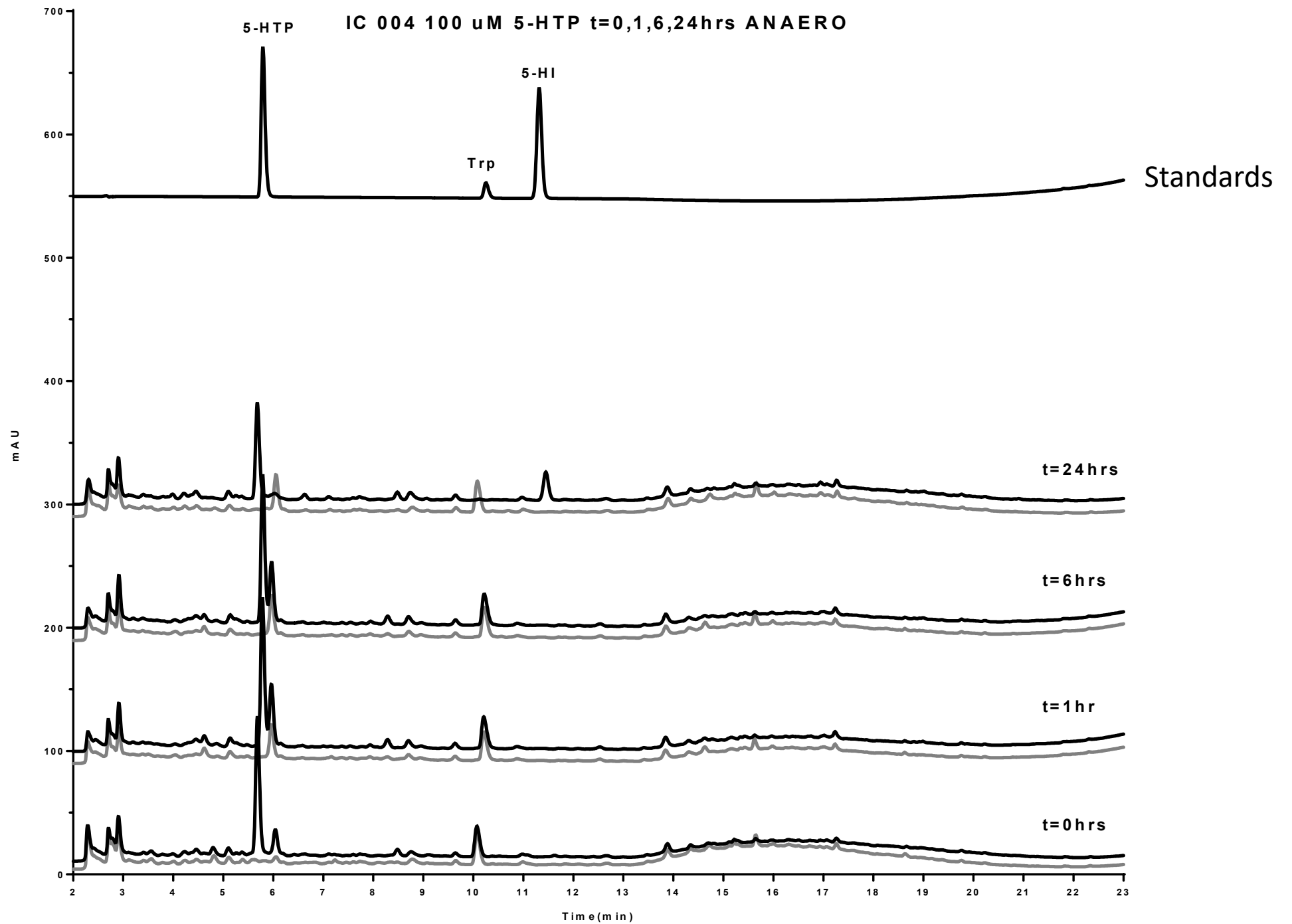

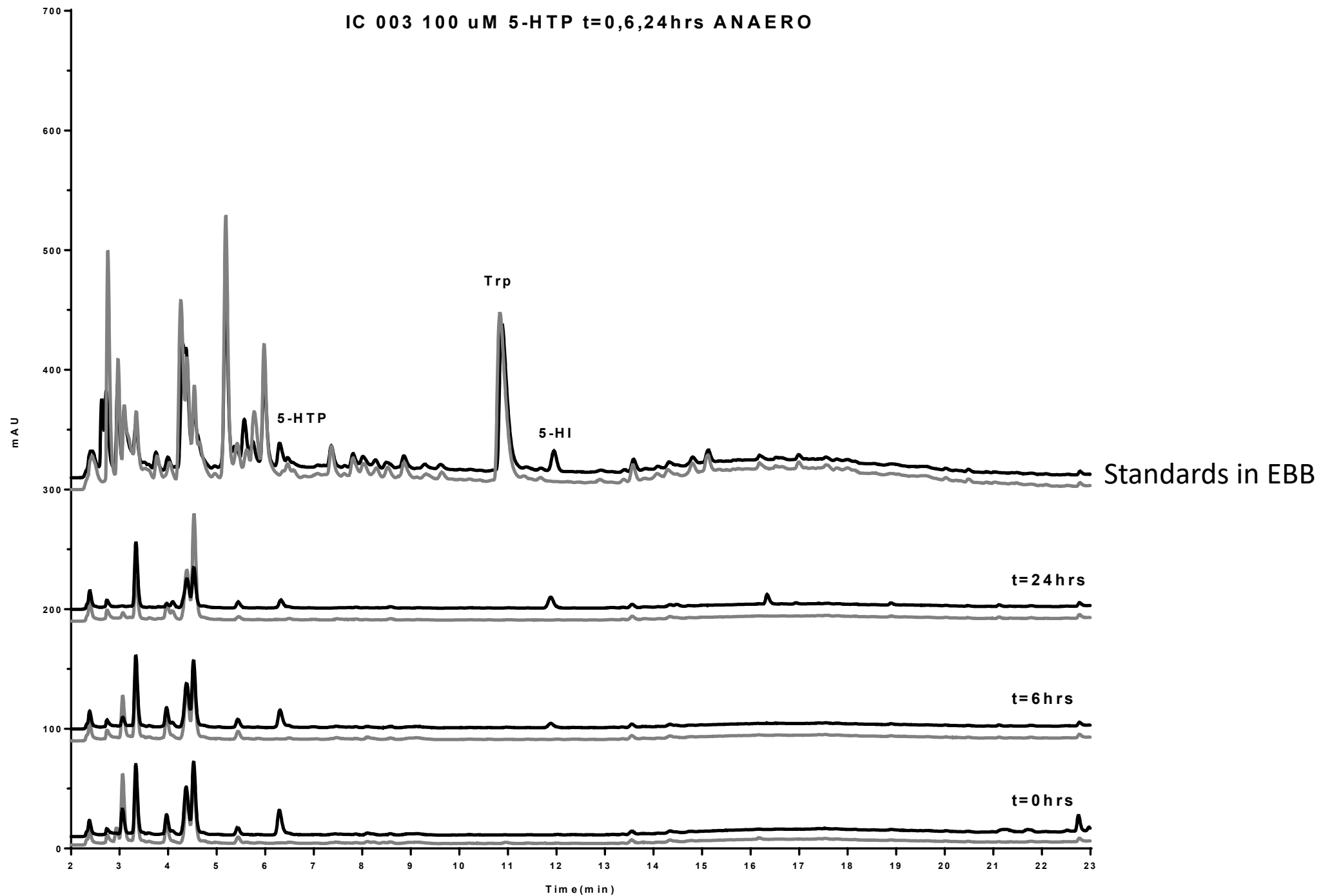

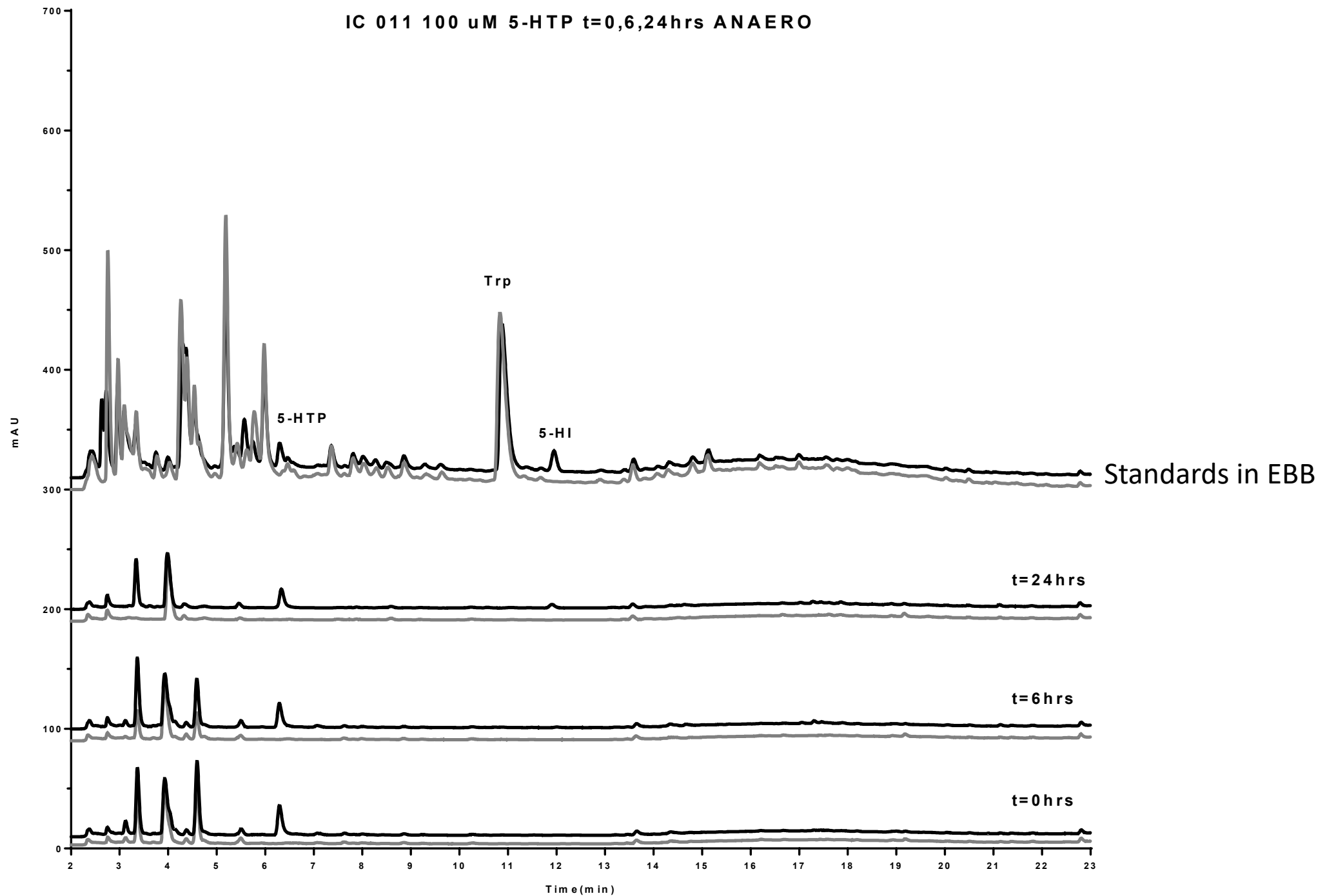

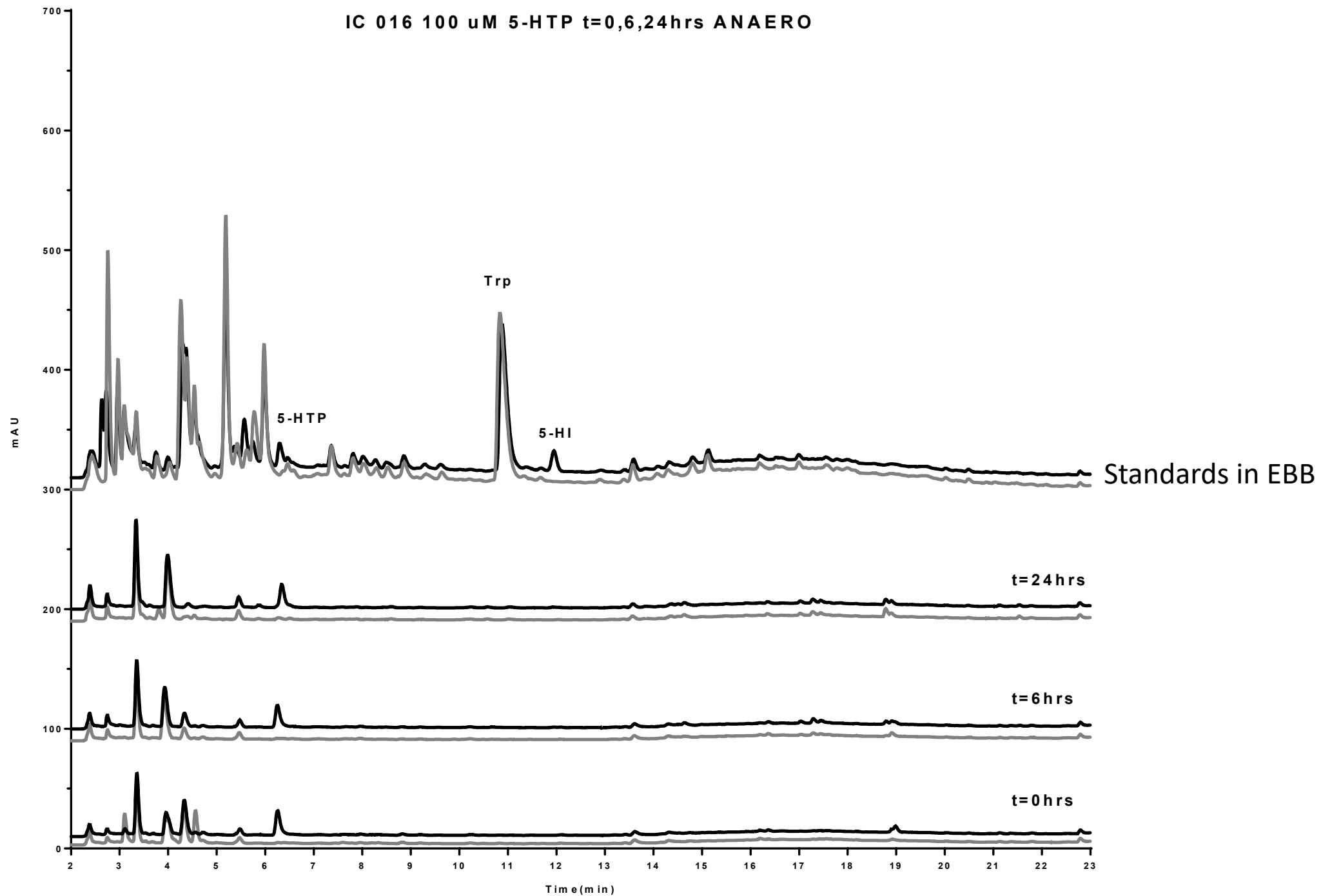

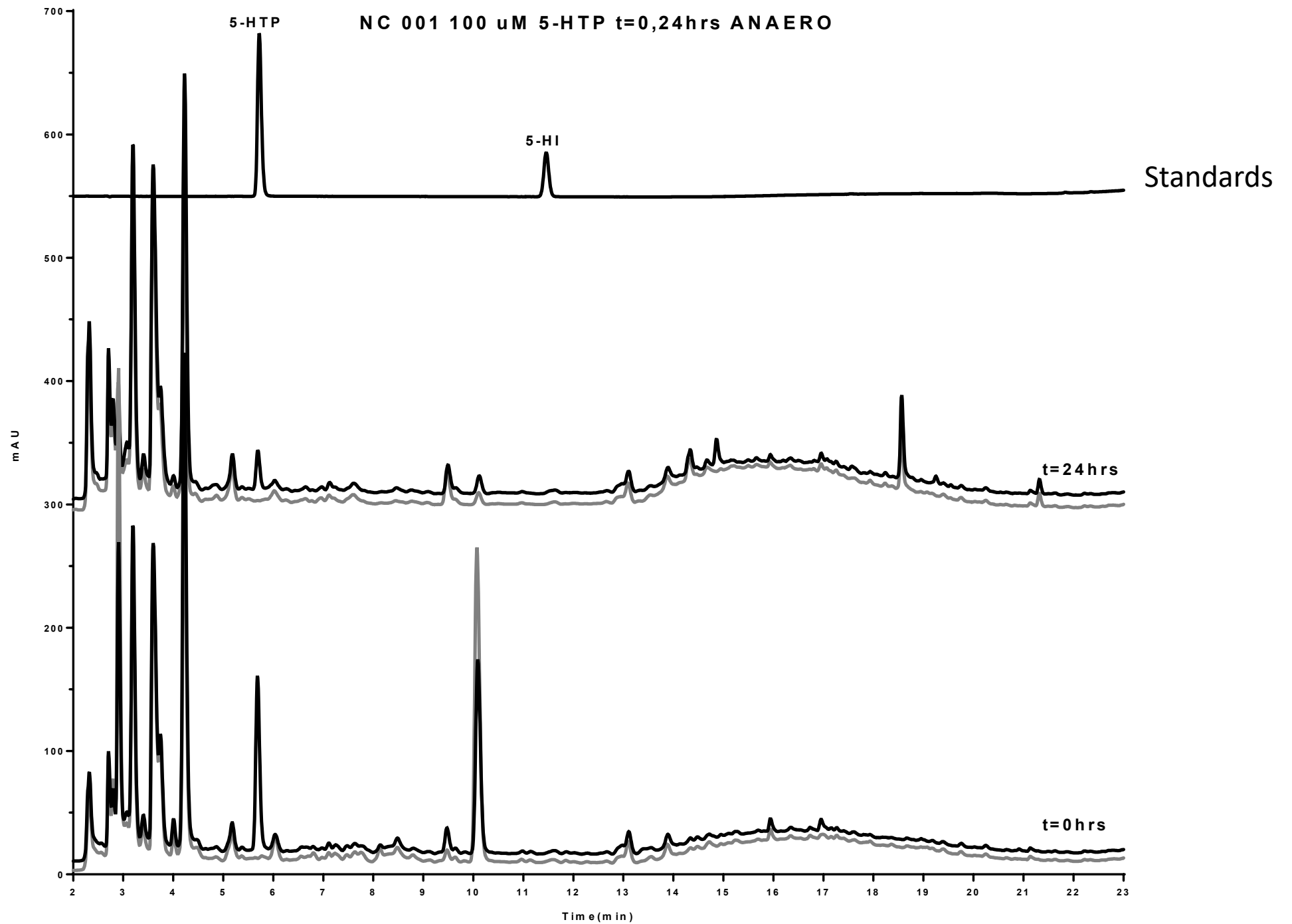

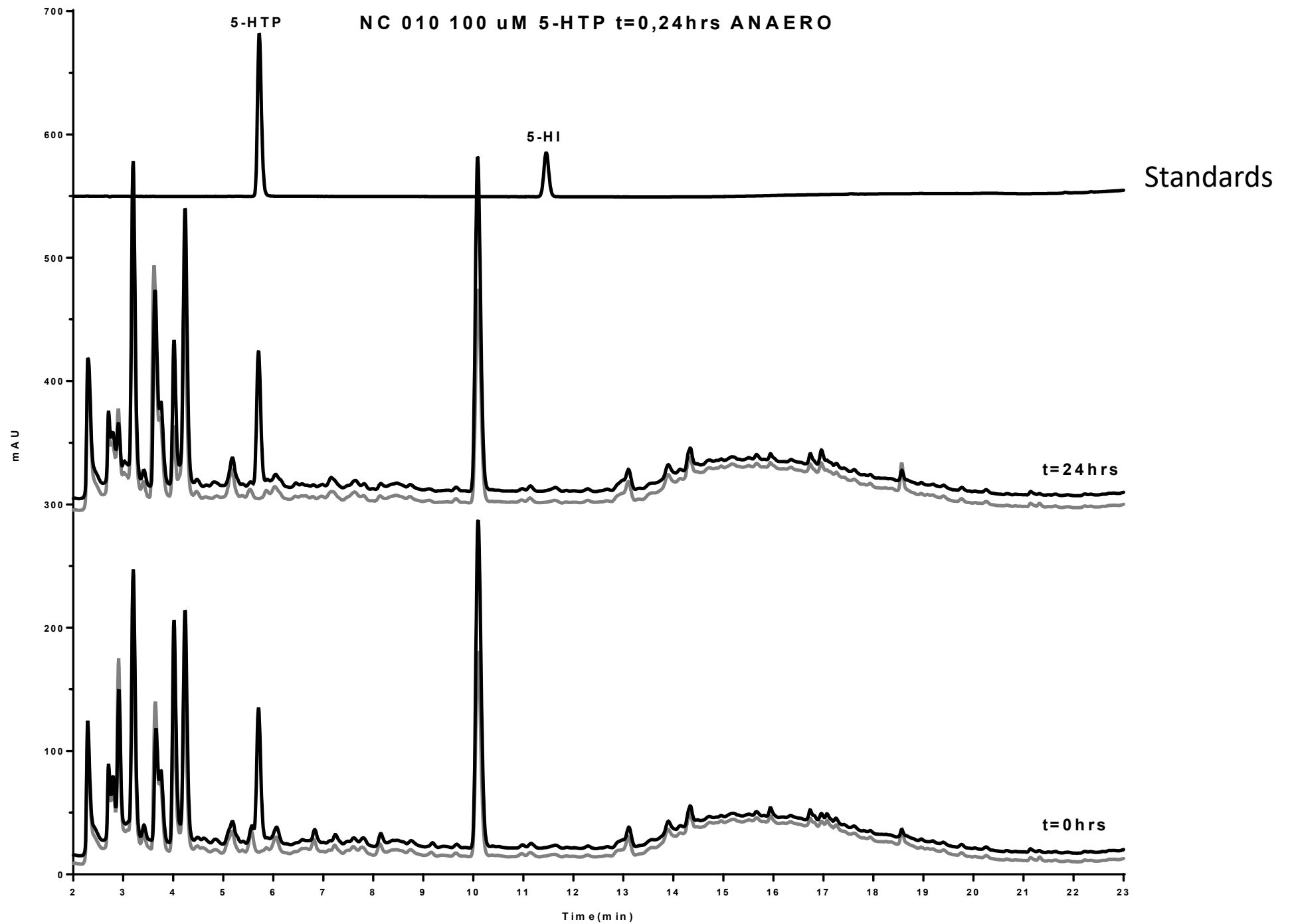

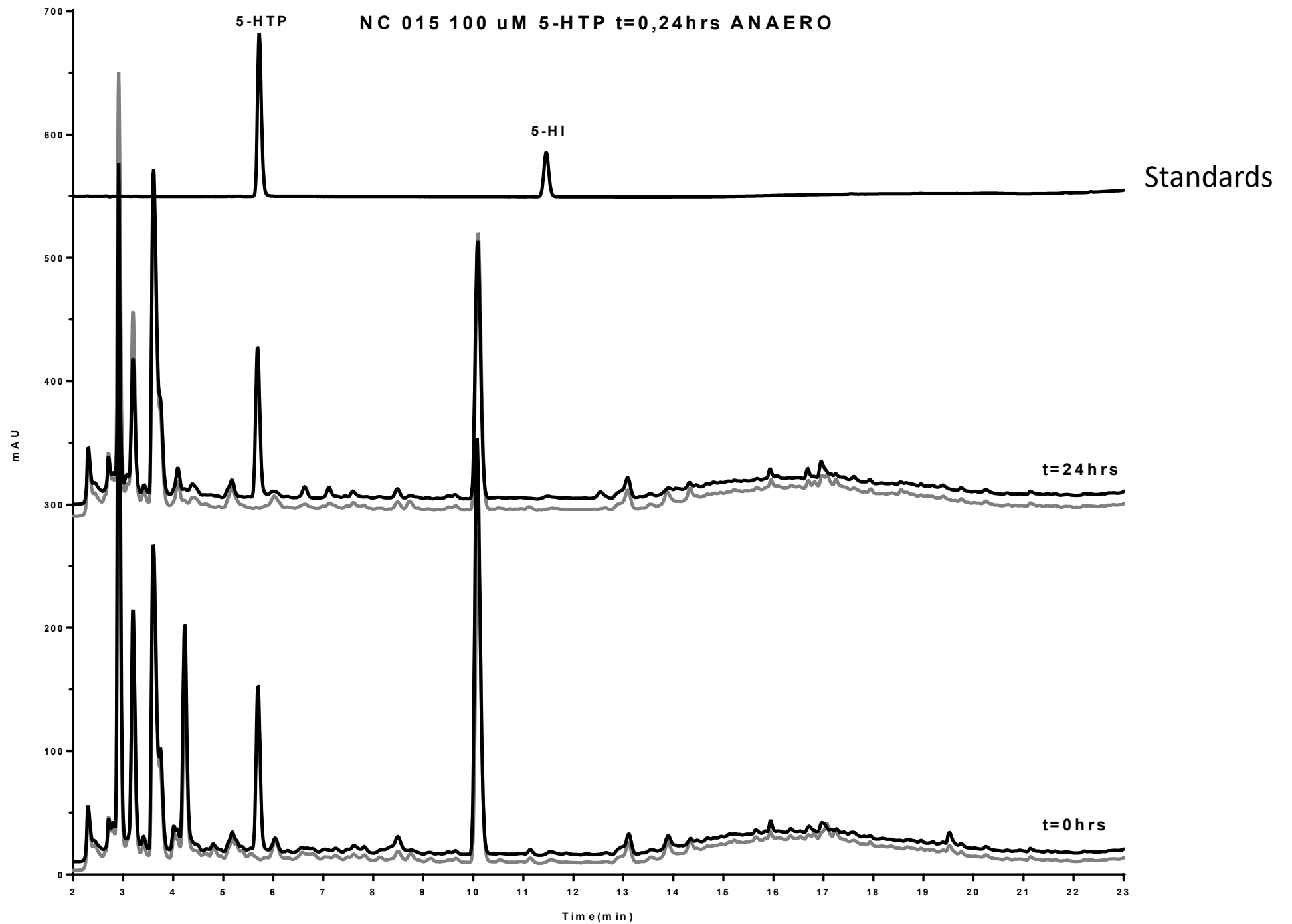

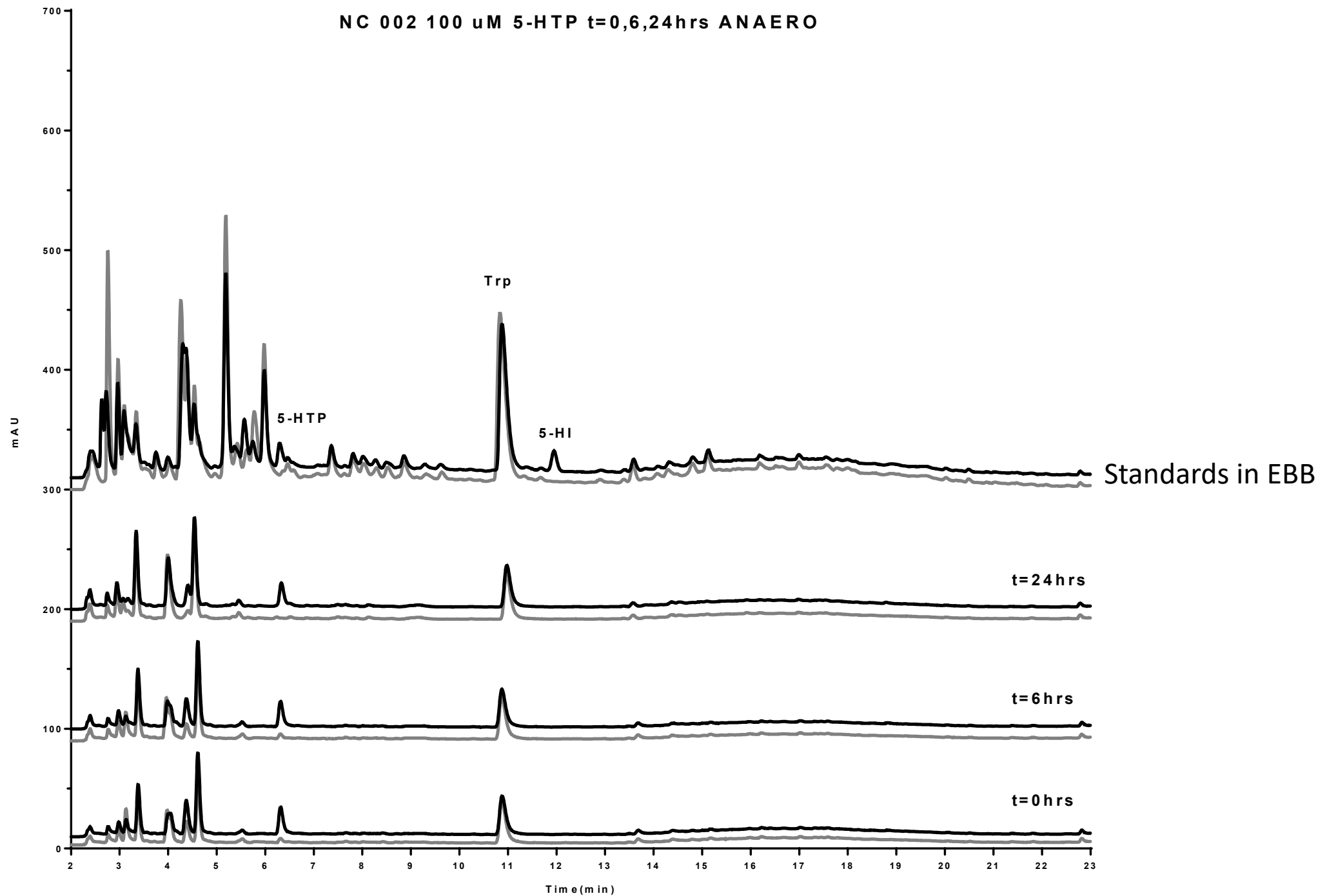

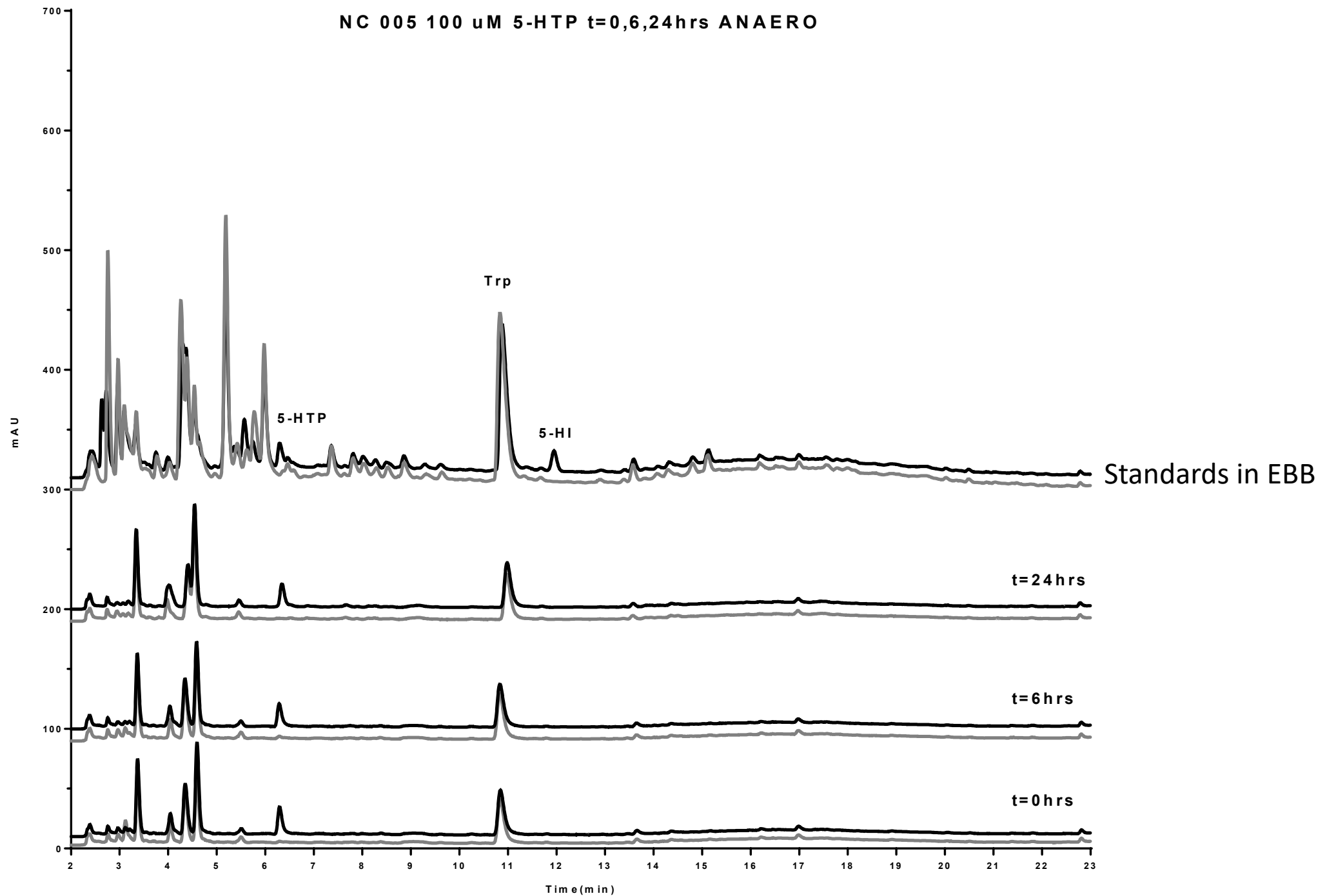

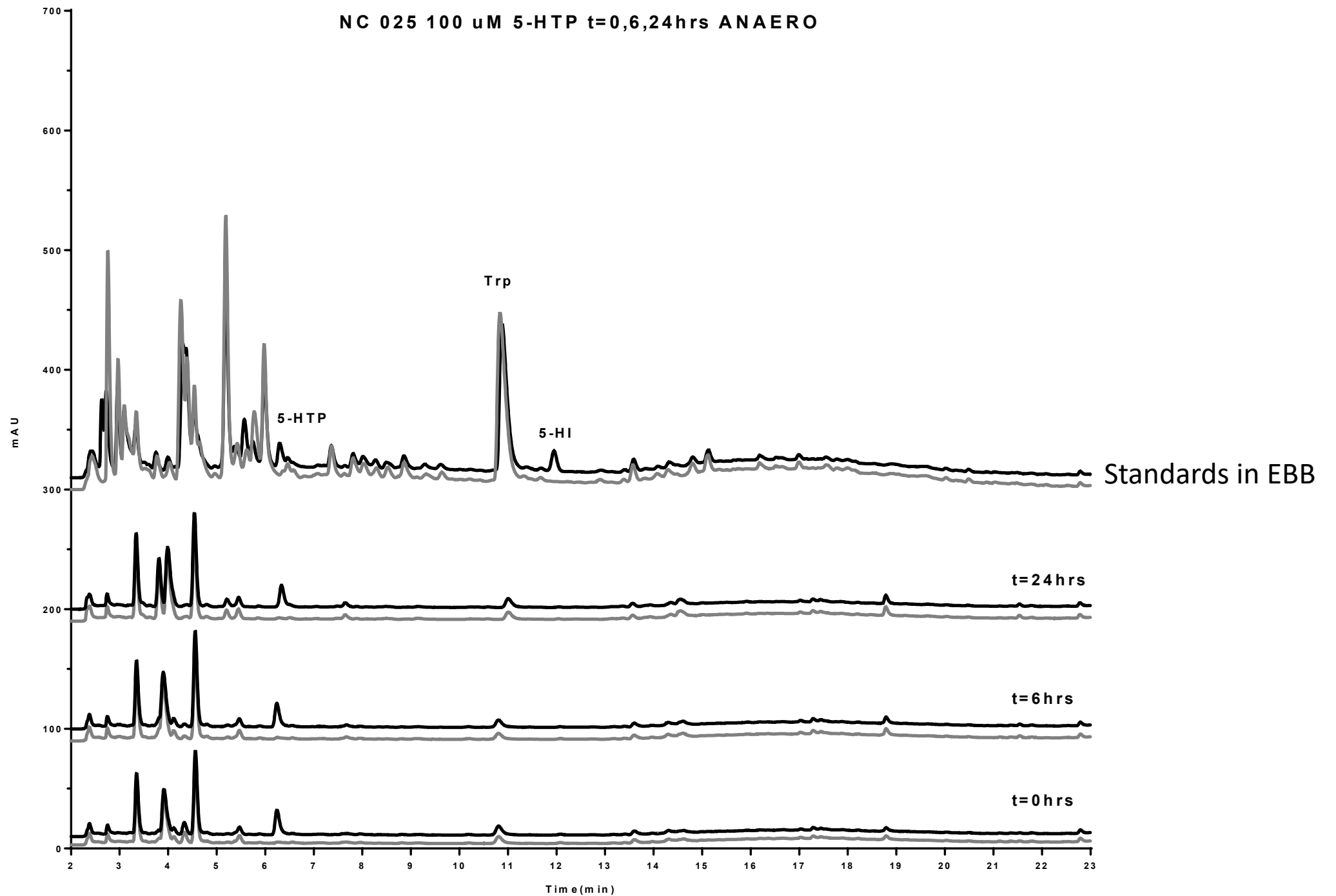

Supplement: S1 Raw images — (PDF) [file pbio.3001070.s010.pdf]

*E. coli* BW 25113

W T vs  $\Delta tnaA$

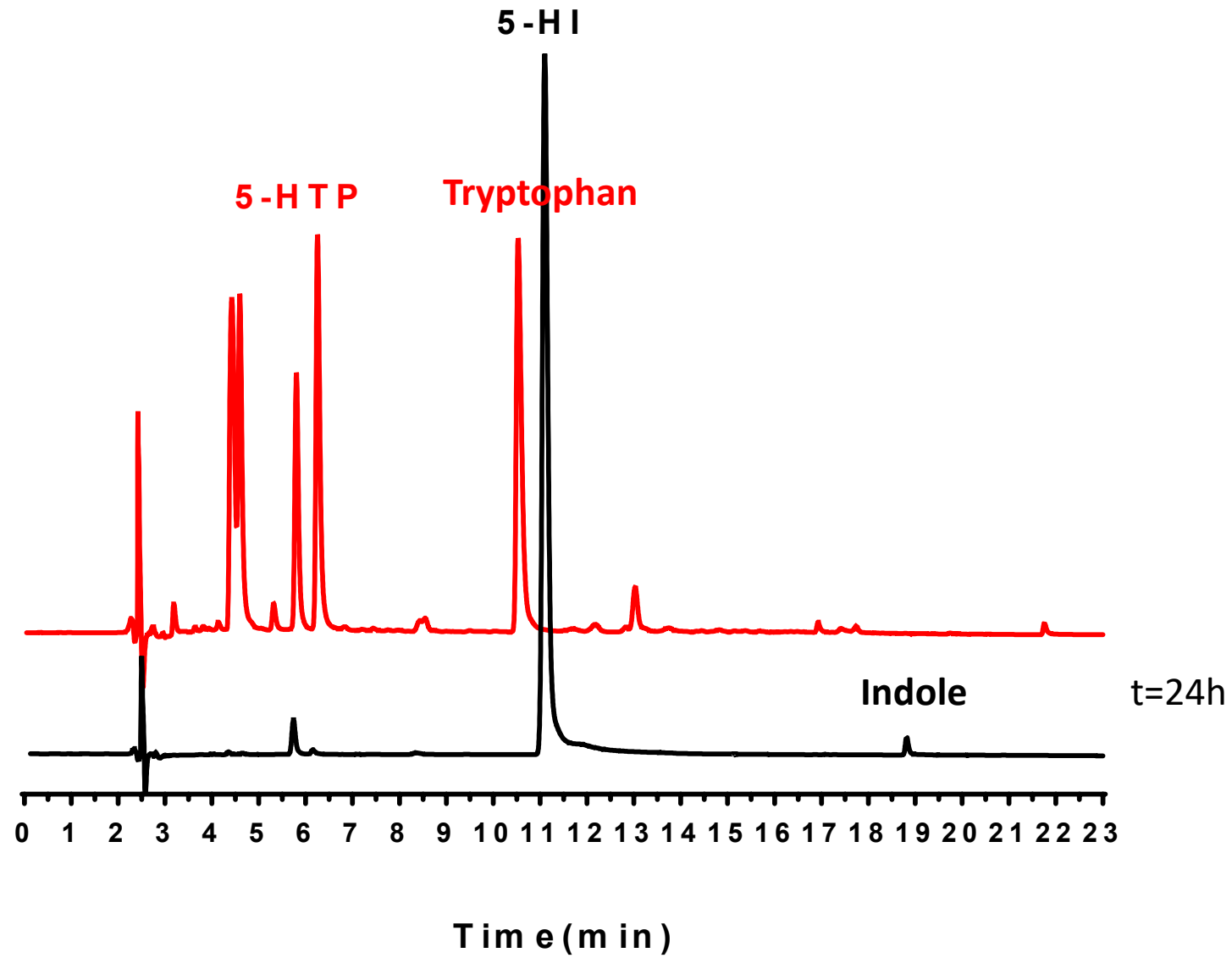

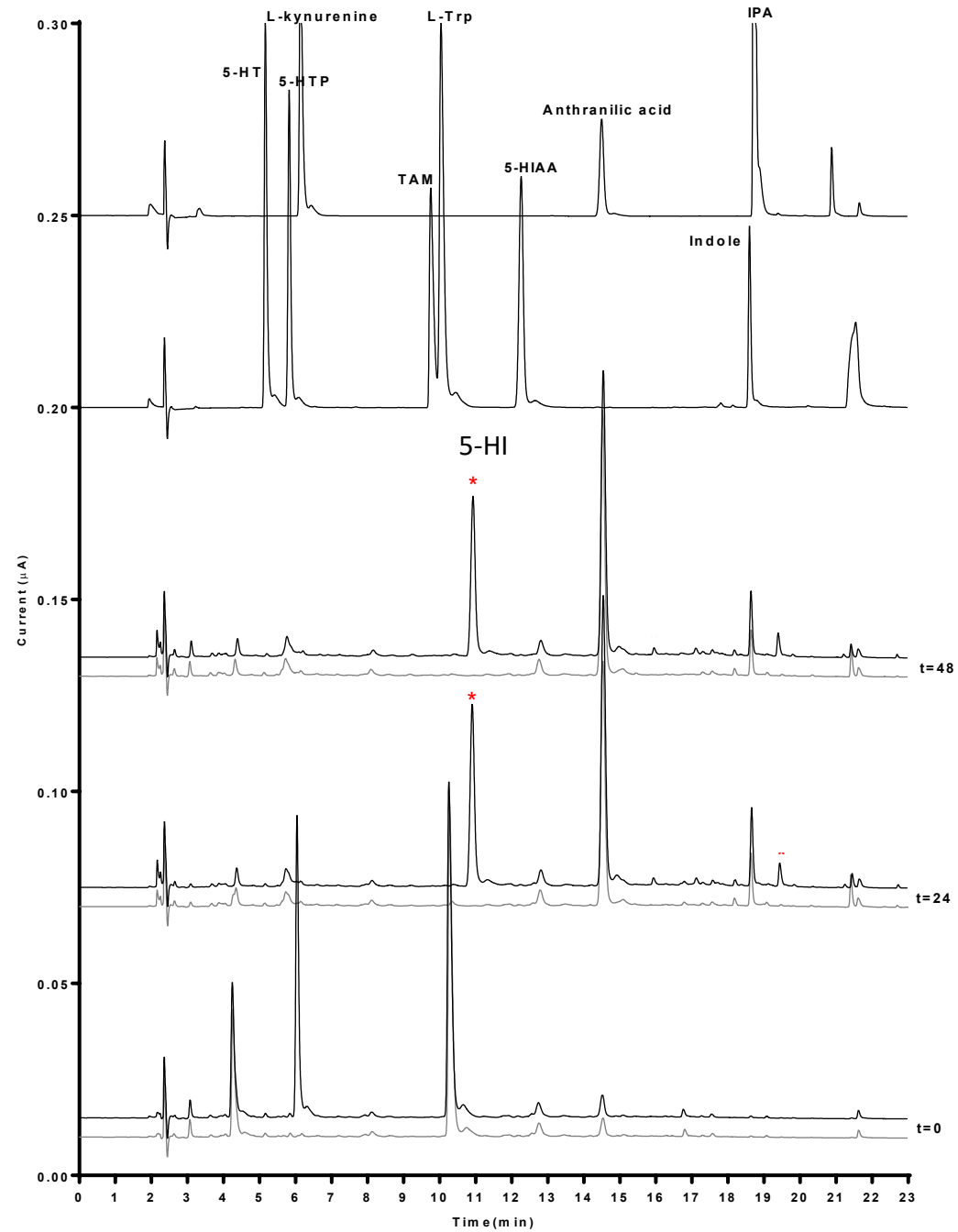

F.sim iae

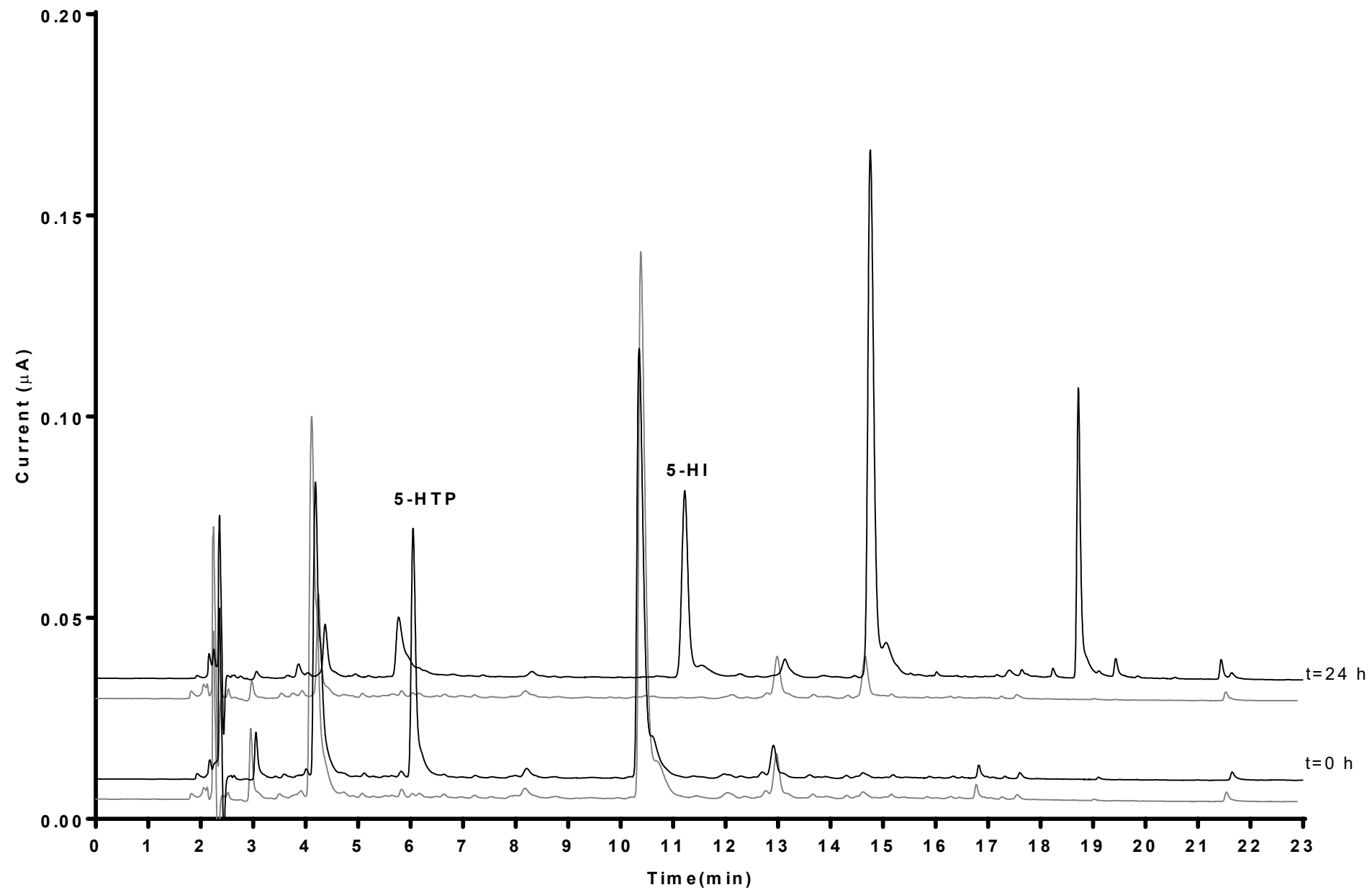

Supplement: S2 Raw images — (PDF) [file pbio.3001070.s011.pdf]

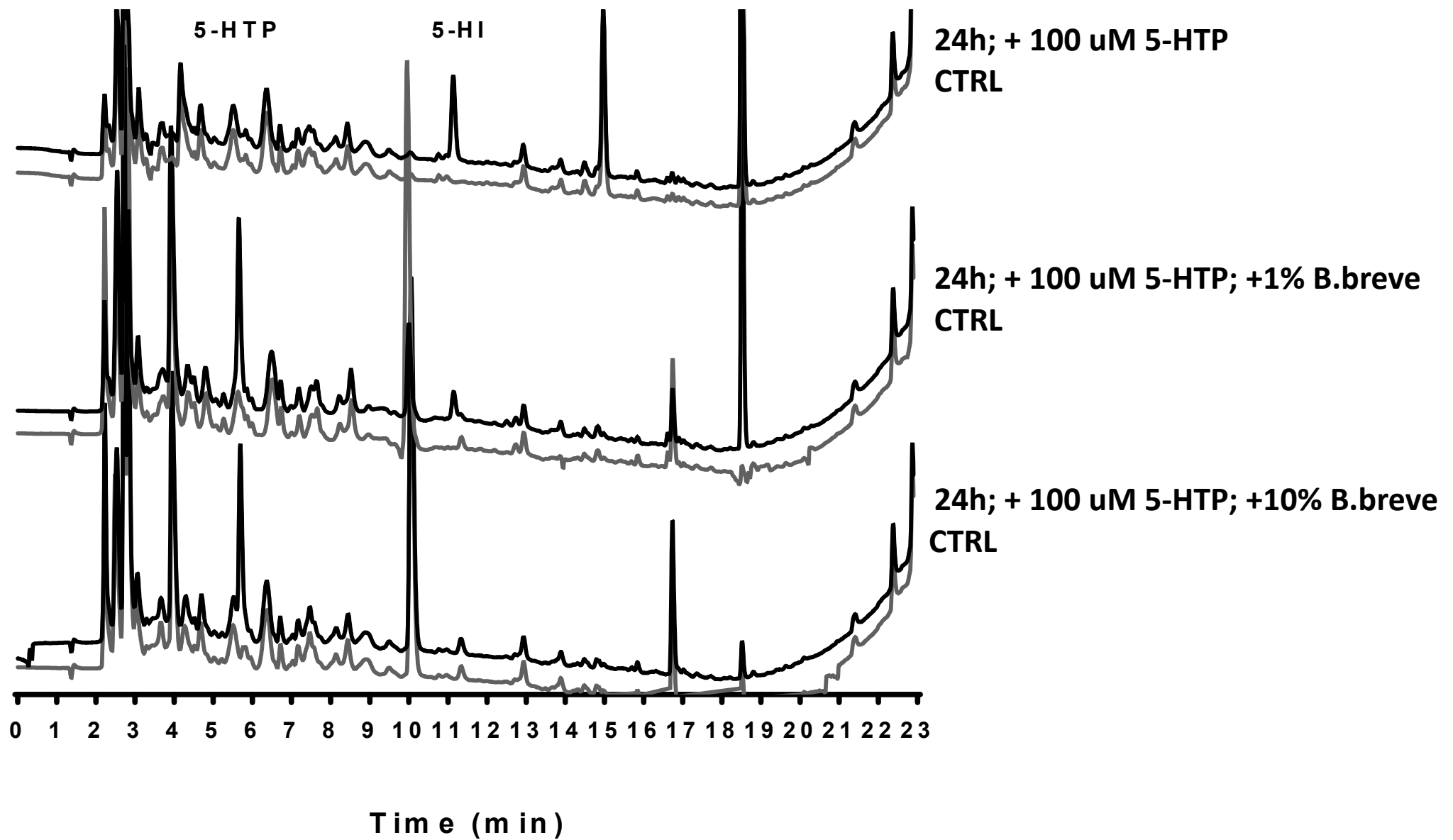

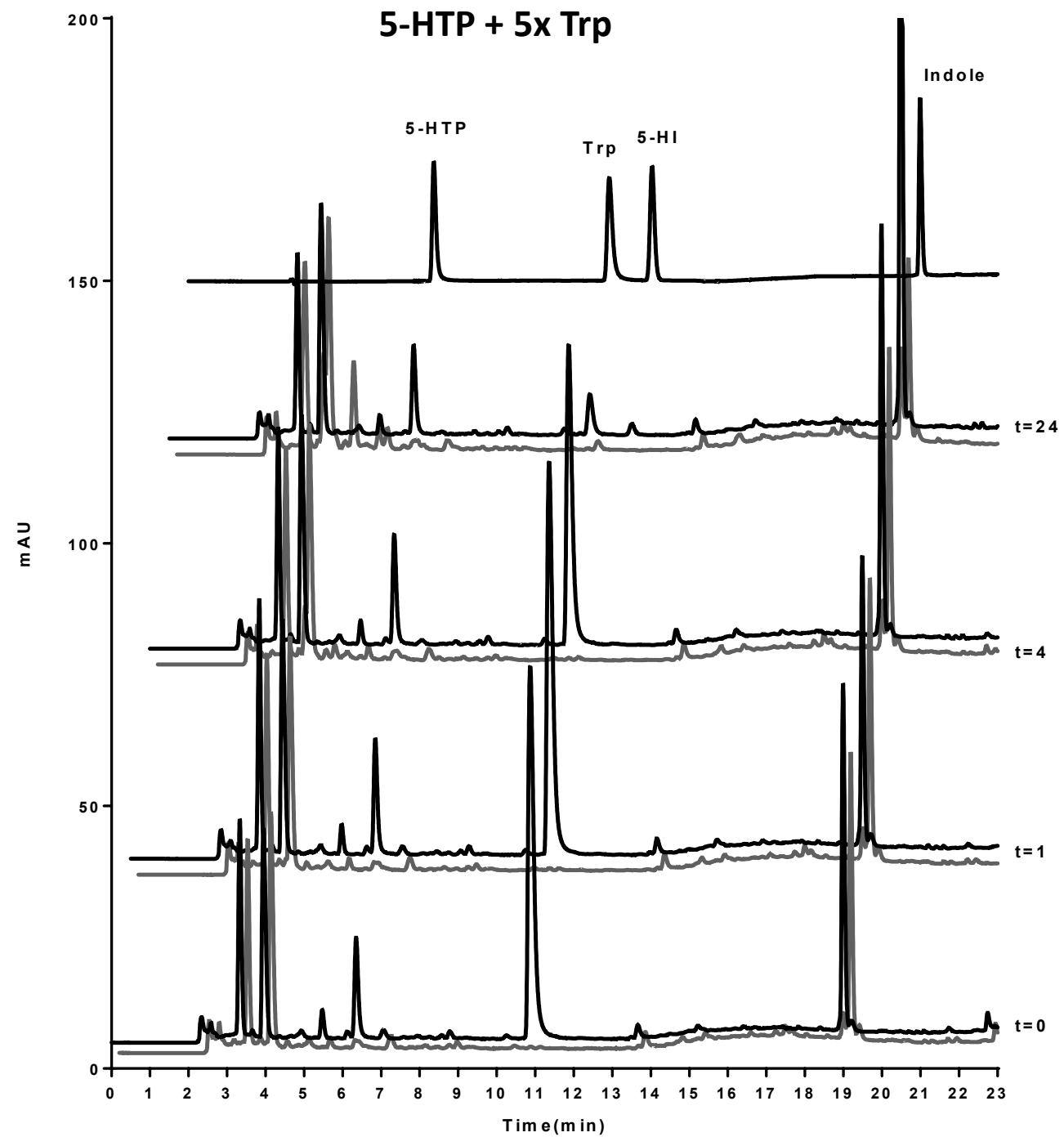

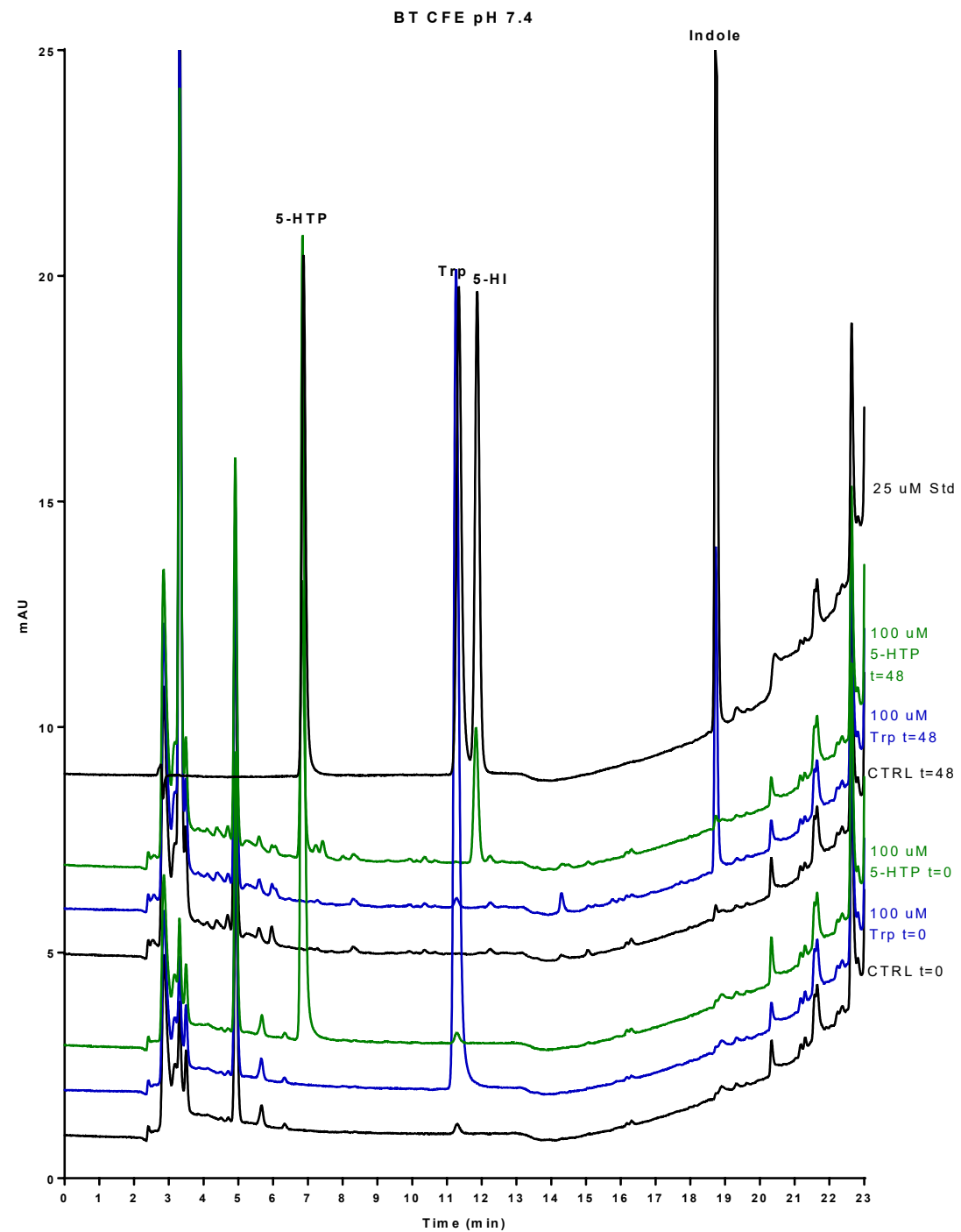

Supplement: S3 Raw images — (PDF) [file pbio.3001070.s012.pdf]
